# Probabilistic neural transfer function estimation with Bayesian system identification

**DOI:** 10.1371/journal.pcbi.1012354

**Published:** 2024-07-31

**Authors:** Nan Wu, Isabel Valera, Fabian Sinz, Alexander Ecker, Thomas Euler, Yongrong Qiu

**Affiliations:** 1 Department of Computer Science, Saarland University, Saarbrücken, Germany; 2 Institute for Ophthalmic Research and Centre for Integrative Neuroscience (CIN), Tübingen University, Tübingen, Germany; 3 Department of Computer Science and Campus Institute Data Science (CIDAS), Göttingen University, Göttingen, Germany; 4 Max Planck Institute for Dynamics and Self-Organization, Göttingen, Germany; 5 Department of Ophthalmology, Byers Eye Institute, Stanford University School of Medicine, Stanford, California, United State of America; 6 Stanford Bio-X, Stanford University, Stanford, California, United State of America; 7 Wu Tsai Neurosciences Institute, Stanford University, Stanford, California, United State of America; Brown University, UNITED STATES OF AMERICA

## Abstract

Neural population responses in sensory systems are driven by external physical stimuli. This stimulus-response relationship is typically characterized by receptive fields, which have been estimated by *neural system identification* approaches. Such models usually require a large amount of training data, yet, the recording time for animal experiments is limited, giving rise to epistemic uncertainty for the learned neural transfer functions. While deep neural network models have demonstrated excellent power on neural prediction, they usually do not provide the uncertainty of the resulting neural representations and derived statistics, such as most exciting inputs (MEIs), from *in silico* experiments. Here, we present a Bayesian system identification approach to predict neural responses to visual stimuli, and explore whether explicitly modeling network weight variability can be beneficial for identifying neural response properties. To this end, we use variational inference to estimate the posterior distribution of each model weight given the training data. Tests with different neural datasets demonstrate that this method can achieve higher or comparable performance on neural prediction, with a much higher data efficiency compared to Monte Carlo dropout methods and traditional models using point estimates of the model parameters. At the same time, our variational method provides us with an effectively infinite ensemble, avoiding the idiosyncrasy of any single model, to generate MEIs. This allows us to estimate the uncertainty of stimulus-response function, which we have found to be negatively correlated with the predictive performance at model level and may serve to evaluate models. Furthermore, our approach enables us to identify response properties with credible intervals and to determine whether the inferred features are meaningful by performing statistical tests on MEIs. Finally, *in silico* experiments show that our model generates stimuli driving neuronal activity significantly better than traditional models in the limited-data regime.

## Author summary

Neural system identification methods learn stimulus-response functions using experimental data to predict responses. These neuronal prediction models demand large amounts of training data, however, the recording time for each experiment is restricted, introducing the uncertainty about the neural features derived from trained models. Here, we present a Bayesian approach incorporating weight uncertainty to identify response functions and show that our method has higher or comparable predictive performance with a higher data efficiency compared to traditional methods using point estimates of model parameters. Additionally, our model provides an effective infinite ensemble to derive neural features, which avoid the idiosyncrasy of a single model. In this way, our method also allows us to estimate the uncertainty of the derived features and to conduct statistical tests on them. Generally, our Bayesian approach enables us to generate many similar stimuli to investigate biological information processing.

## Introduction

Current neural interfaces allow to simultaneously record large populations of neural activity. In sensory neuroscience, such ensemble responses are driven by external physical stimuli (e.g., natural images), and their relation has been characterized by tuning curves or receptive fields (RFs; [1]). Such stimulus-response functions have been estimated by *neural system identification* methods (reviewed in [2]). Classically, these approaches used a linear-nonlinear-Poisson (LNP) model or variants of it [3–6] to predict responses to unseen stimuli such as white noise and natural images [7, 8]. More recently, deep neural networks (DNNs) with multiple layers of non-linear processing have shown great success for learning neural transfer functions along the ventral visual stages from retina [9–11] and primary visual cortex [12–15] to higher visual areas [16, 17]. Moreover, through *in silico* experiments, these models are able to generate specific stimuli to control neural activity and identify novel neuronal properties from a high-dimensional space [18–22]. For example, closed-loop paradigms show that performing gradient ascent on a deep model can yield most exciting inputs (MEIs) to drive a neuron’s activity optimally [18–20, 23].

Yet, these system identification approaches demand significant amounts of stimulus-response pair data for the model training, given the high dimensional stimulus space and the non-linear neural transformations [9, 15, 24, 25]. Due to limited recording time for each experiment, the amount of data for fitting these models is restricted introducing epistemic uncertainty about the learned stimulus-response function. To estimate this uncertainty, traditional LNP methods obtain full posterior distribution of model parameters by leveraging a Bayesian framework to provide confidence intervals for the estimated RFs [5, 24, 26–28]. However, DNN models rarely consider the uncertainty of the neuronal properties that are recovered from *in silico* experiments. Typically, MEIs are computed on an ensemble of models to avoid the idiosyncrasy of a single model. Utilizing a full posterior provides us with an effectively infinite ensemble through sampling. Additionally, it is important to quantify the uncertainty of derived features as we are interested in whether they are biologically meaningful. Consider a scenario: we observe elements in the surround, but they are faint. This prompts the question: Are they real? With a single MEI, we cannot answer that; however, using a model equipped with uncertainty, we can assess whether the posterior significantly deviates from zero.

**Fig 1:**
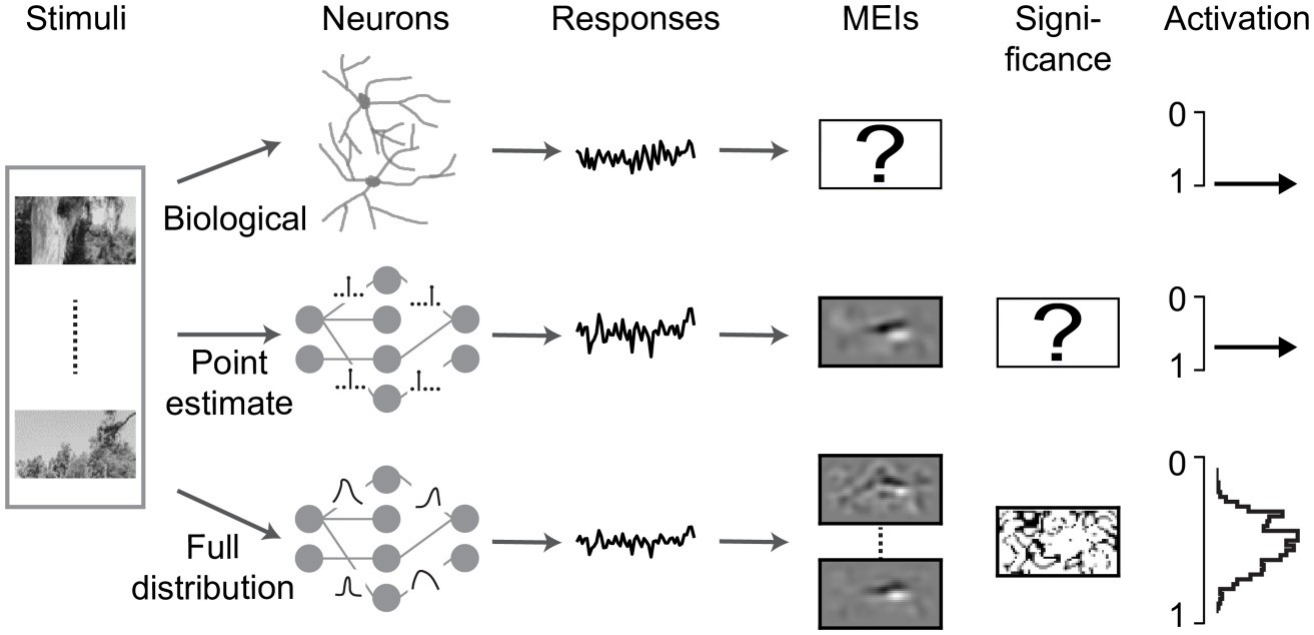
Schematic of neural system identification for predicting responses. Biological neurons (top row; second column) respond to visual stimuli (first column) distinctly (third column), with an unknown MEI (fourth column) driving a cell with optimal activation (sixth column). Traditional system identification methods (center row) learn stimulus-response function and yield a MEI with unknown statistics (fifth column). Bayesian approaches (bottom row) learn distributions of model parameters to predict neuronal responses, yielding infinite MEIs, whose significance map can be computed by sampling from posterior, to drive a neuron with credible intervals.

Here, we propose a Bayesian system identification approach to estimate response features of neurons with uncertainties (Fig 1). We test whether incorporating uncertainties by learning the full distribution of model parameters is beneficial for learning neural representations. To this end, we build a DNN model to predict responses to unseen visual stimuli by using variational inference to estimate the distribution of network weights, i.e., Bayes by Backprop [29–32].

Our contributions are: (1) We incorporate weight variability in deep neural networks for identifying neural response functions with uncertainty and extend the Bayes by Backprop with a hyperparameter which effectively adjusts the sparsity of model parameters. (2) We apply our Bayesian models on different experimental datasets and find that our method can achieve higher or comparable performance on neural prediction, with a much better data efficiency, compared to Monte Carlo dropout methods and traditional models using point estimates of the model parameters. (3) Our approach with full posterior allows to estimate neural features with credible intervals and run statistical test for the derived MEIs, bypassing the idiosyncrasy of a single model. (4) Finally, simulation experiments demonstrate that the variational model yields stimuli that drive neuronal activation better than the traditional models in the condition of limited training data. This supports that weight uncertainty, as implemented in our model, may contribute to a more efficient identification of non-linear neuronal response functions.

## Materials and methods

### Dataset

We tested our method on two publicly available datasets.

The first dataset contains calcium signals driven by static natural gray-scale images for neurons in primary visual cortex (V1) of mice [12]. We used 103 neurons from the first scan field, whose single-trial responses to 1,600 images for training models and 200 for tuning hyperparameters. Then we used the mean of response repeats to 50 test images for evaluating models.

The second dataset comprises Ca^2+^ responses to natural green/UV images (36x64 pixels) for neurons in mouse V1 [21]. We selected the natural stimuli that were presented in both UV and green channels and used the neurons whose quality index (*QI* = Var[E[*C*]_*r*_]_*t*_/E[Var[*C*]_*t*_]_*r*_, time samples *t* and repetitions *r*, a response matrix *C* with a shape of *t* × *r*, E[*X*]_*d*_ and Var[*X*]_*d*_ denoting the mean and variance along the dimension *d* of *X*, respectively) of 10-repeat test responses were larger than 0.3. In this way, we obtained 161 neurons from one scan field, whose single-trial responses to 4,000 images for training and 400 for validation. Then we used mean of response repeats to 79 test images for evaluation.

### Models

#### Variational model

DNN for system identification can be seen as a probabilistic model: given the training data $D={\left(x_{i},y_{i}\right)}_{i}$ where **x**_*i*_ is an input (such as natural images) and **y**_*i*_ is the output (such as neural responses), we aim to learn the weights **w** of a network which can predict the output for the unseen stimuli (Fig 1). Compared to a traditional method using point estimates of the weights, Bayesian approaches learn full distributions of these **w**. Estimating the full posterior distribution of the weights P(w|D) given the training data is usually not feasible. An alternative is to approximate P(w|D) by a new distribution *q*(**w**|*θ*) whose parameters *θ* are trained to minimize the distance between the proxy and the true posterior, which is called variational inference [29–32]. Usually we use Kullback-Leibler (KL) divergence as a measure of distance between two distributions:

$$\begin{array}{c} \theta^{*}=\underset{\theta }{\text{arg}\;\text{min}}KL\left[q\left(w|\theta \right)||P\left(w|D\right)\right] \end{array}$$ (1)

$$\begin{array}{c} =\underset{\theta }{\text{arg}\;\text{min}}KL\left[q\left(w|\theta \right)||P\left(w\right)\right]-E_{q(w|\theta )}\left[\text{log}\;P\left(D|w\right)\right] \end{array}$$ (2)

The optimization function can be viewed as a trade-off between the distance between the variational posterior and the selected prior and the likelihood cost. We can view it as a constrained optimization problem as [33]:

$$\begin{array}{c} \underset{\theta }{\text{arg}\;\text{min}}E_{q(w|\theta )}\left[\text{log}\;P\left(D|w\right)\right]\;\;\;\;\text{subject}\;\text{to}\;\;KL\left[q\left(w|\theta \right)||P\left(w\right)\right]<\epsilon \end{array}$$ (3)

Here *ϵ* represents the specific distance between the variational posterior and the prior. According to KKT conditions [34] and non-negative properties of KL divergence, we get:

$$\begin{array}{c} F=E_{q(w|\theta )}\left[\text{log}\;P\left(D|w\right)\right]-\beta_{v}\left(KL[q\left(w|\theta \right)||P\left(w\right)]-\epsilon \right) \end{array}$$ (4)

$$\begin{array}{c} \geq E_{q(w|\theta )}\left[\text{log}\;P\left(D|w\right)\right]-\beta_{v}KL\left[q\left(w|\theta \right)||P\left(w\right)\right] \end{array}$$ (5)

where *β*_*v*_ is non-negative and represents a Lagrangian multiplier. So the final loss function for the model is:

$$\begin{array}{c} L=\beta_{v}KL\left[q\left(w|\theta \right)||P\left(w\right)\right]-E_{q(w|\theta )}\left[\text{log}\;P\left(D|w\right)\right] \end{array}$$ (6)

$$\begin{array}{c} \approx \sum_{i=1}^{n}\;\beta_{v}\left(\text{log}\;q\left(w^{\left(i\right)}|\theta \right)-\text{log}\;P\left(w^{\left(i\right)}\right)\right)-\text{log}\;P\left(D|w^{\left(i\right)}\right) \end{array}$$ (7)

Eq (7) is a result of Monte Carlo sampling *n* instances **w**^(*i*)^ from *q*(**w**|*θ*) because we can not calculate (6) directly. Note that the use of *β*_*v*_ is equivalent to a common operation of tempering likelihood in KL-weighted evidence lower bound [35–37].

Here, we implemented convolutional neural networks (CNNs) for all experiments. For a CNN using variational inference on model weights (variational model), we picked independent Gaussian distributions for the variational posterior and a scale mixture of two Gaussians for the prior [32]. The log posterior was defined as

$$\text{log}\;q\left(w|\theta \right)=\sum_{k=1}\text{log}\;N\left(w_{k}|\mu^{k},{\left(\sigma^{k}\right)}^{2}\right)$$

where *w*_*k*_ denotes *k*th weight of the neural network and (*μ*^*k*^, *σ*^*k*^) are the posterior parameters *θ*^*k*^. We omitted the superscript *k* for *μ* and *σ* in other formulas to maintain simplicity when there is no ambiguity. To keep *σ* non-negative, we parameterised it using *σ* = log(1 + exp(*ρ*)). We selected the log prior

$$\text{log}\;P\left(w\right)=\sum_{k=1}\text{log}\;\left(\pi N\left(w_{k}|0,\sigma_{1}^{2}\right)+\left(1-\pi \right)N\left(w_{k}|0,\sigma_{2}^{2}\right)\right)$$

where *π* is a mixture component weight (0 ≤ *π* ≤ 1) [32, 38]. This prior, compared to a single Gaussian distribution, encourages sparseness in learned kernels, reminiscent of neural representations in visual systems [39–42]. The likelihood loss depends on the specific task of the network. For neural system identification, we use Poisson loss $-\text{log}\;P\left(D|w\right)=\sum_{l}\;{\hat{r}}_{l}-r_{l}\;\text{log}\;{\hat{r}}_{l}$, where *l*, ${\hat{r}}_{l}$ and **r**_*l*_ denote neuronal index, prediction responses and true responses, respectively.

#### Baseline and control models

We used a CNN without any regularization as a baseline model and used a CNN with L2 regularization in each convolutional layer and L1 regularization in fully connected layer (L2+L1) as a control model. We adopted an ensemble of L2+L1 models with different initialization seeds as a second control model, whose predicted responses are the average of five model outputs. To examine the contribution from weight uncertainties, we built a maximum a posteriori (MAP) model which contains prior and likelihood terms in Eq (7) as loss functions. Additionally, as a fourth control, we adopted a CNN with Monte Carlo dropout for probabilistic prediction; it used the same dropout rate for each model layer and in both training and test stages [43, 44].

#### Model details

The CNN model for the first dataset shared by Antolik and colleagues consisted of a convolutional layer (24x1x9x9, output channels x input channels x image width x image height), a rectified linear unit (ReLU) function, another convolutional layer (48x24x7x7, output channels x input channels x image width x image height), another ReLU function, and—after flattening all dimensions—one fully connected (FC) layer (103x13872, output channels x input channels), followed by an exponential function (S1 Appendix). We used stride = 1 and no padding for both convolutional layers. We trained the six models and tuned their respective hyperparameters. For the variational one, we tested different parameters for prior distribution on validation data, such as *π* = 0 or *π* = 0.5, *σ*_1_ = 1 or *σ*_1_ = 100, *σ*_2_ = exp(−3) or *σ*_2_ = exp(−6), and found that a scale mixture of two Gaussians had similar predictive performance, higher than one Gaussian distribution. As the predictive performance was similar for distinct priors on model layers, we used the same prior distribution with parameters *π* = 0.5, *σ*_1_ = 1, *σ*_2_ = exp(−6) for all layers. We also examined the number of Monte Carlo sampling times for model training and found that the predictive performance was similar for different numbers. Therefore, we used 1 or 2 sampling times for all model training.

The CNN model for the second dataset shared by Franke and colleagus contained a convolutional layer (48x2x9x9), a ReLU function, another convolutional layer (48x48x7x7), another ReLU function, and one FC layer (161x52800), followed by an exponential function. We used stride = 1 and no padding for both convolutional layers.

### Training and evaluation

We trained all models with a learning rate of 0.0003 for a maximum of 200 epochs using the Adam optimizer [45]. We computed linear correlation (correlation coefficient, CC) between predicted and recorded responses, which was used to evaluate models on validation or test data. We tuned model hyperparameters and selected the ones as well as the respective epoch number with the best predictive performance on validation data. We also evaluated models on test data using root mean square error (RMSE) and log likelihood, however, similar to other studies on neural prediction [9, 10, 12, 15], we primarily used CC for analysis. To keep the comparison fair, the test models shared similar network architecture for each dataset, except that the dropout model featured dropout layers.

For each trained model, we estimated MEIs of all neurons by running gradient ascent on a random input image for 100 steps with a learning rate of 10 and we picked the stimulus with the highest activity [20, 46]. All generated MEIs had the same mean and standard deviations as the training images. For the two probabilistic (variational and dropout) models, we ran the estimation for 100 times with Monte Carlo sampling, hence, we got 100 MEIs (matrix *C*) for each recorded neuron. Note that we fixed the random seed/state for each sampling, in this way, model weights did not change stochastically during the iterative generation of each MEI. We defined MEI variance of one neuron as MEI variance = E[Var[*C*]_*s*_]_*hw*_ (sampling times *s*, stimulus height *h*, stimulus width *w*, and *C* with a shape of *s* × *h* × *w*). The overall MEI variance for a model was an average of MEI variances for the recorded neurons.

In *in silico* experiments, to measure the activation distribution of MEIs yielded from variational models for a neuron, we estimated 100 MEIs by sampling and one mean MEI by using the weight mean *μ* from each seed. So we had 505 MEIs for five random seeds with one additional MEI which was the mean of the five mean MEIs, in total 506 MEIs. For L2+L1 models, we estimated five MEIs from different random seeds and also got one by averaging across these MEIs, in total 6 MEIs.

## Results

### *β*_*v*_ balances model capacity and data likelihood

Compared to a conventional evidence lower bound in Eq (2), we used a Lagrangian multiplier *β*_*v*_ in (7) by borrowing the idea of constrained optimization from [33]. In this way, Blundell and colleagues’ work can be seen as a special case of *β*_*v*_ = 1.0 [32]. We first analyzed the possible roles of *β*_*v*_. We investigated it from the perspective of information theory, given that Eq (7) has a similar form to the objective functions in deep variational information bottleneck [47, 48] and *β*-VAE [33, 49].

The training objective jointly minimizes the KL divergence between the posterior *q*(**w**|*θ*) and the prior *P*(**w**) and maximizes the data likelihood under the distribution *q*(**w**|*θ*). The distribution distance becomes zero when *q*(**w**|*θ*) = *P*(**w**). The prior comprises a mixture of two Gaussian distributions centered at zero, with one exhibiting a relatively small standard deviation (e.g., a distribution with *π* = 0.5, *σ*_1_ = 1, *σ*_2_ = exp(−6)). Large *β*_*v*_ downweighs the log likelihood $E_{q(w|\theta )}\left[\text{log}\;P\left(D|w\right)\right]$. In the extreme case of very large *β*_*v*_, the variational posterior *q*(**w**|*θ*) converges towards the prior distribution *P*(**w**). However, given that the posterior is restricted to a single Gaussian and that minimizing the reverse KL results in mode-seeking and zero-forcing, the weights **w** of the posterior will largely be forced to zeros. In such a case, the model has very sparse parameters but very limited expressive power. Therefore, *β*_*v*_ can be interpreted as a coefficient to adjust the sparsity of model parameters for fitting the data.

**Fig 2:**
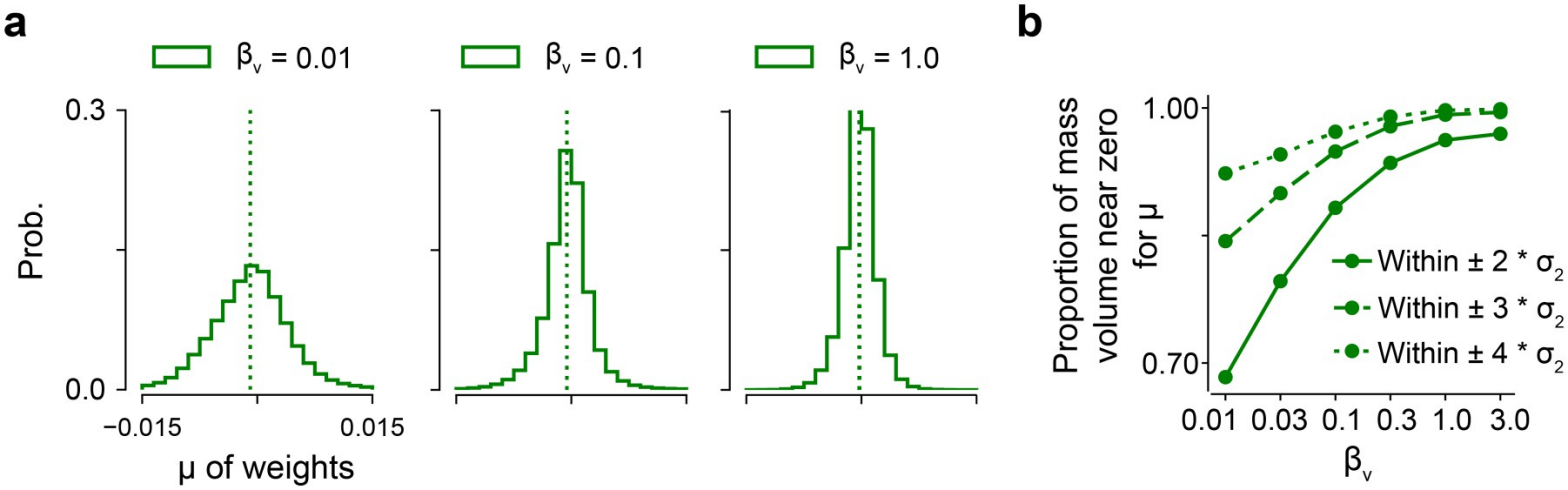
Hyperparameter *β*_*v*_ for regulating weight sparseness. **(a)** Distribution of the means (*μ*) of model weights for different *β*_*v*_ values. Dotted lines indicate distribution means. **(b)** Ratio of mass volume near zero for the distributions in (a). Note that with our setup, if we use a mixture of two Gaussians for the posterior, we would not observe a higher weight sparseness with a larger *β*_*v*_; rather, we would observe a wider distribution of model parameters.

Empirically, we measured the sparsity of the model weight means (*μ*) for different *β*_*v*_ values on the dataset 1 shared by Antolik and colleagues [12]. We computed the proportion of mass volume near zero, e.g., within a certain threshold, for the weight means. We used thresholds of ±2 * *σ*_2_, ±3 * *σ*_2_ and ±4 * *σ*_2_, and observed an increase of the ratio with the rise of *β*_*v*_ for the three conditions, indicating an increase of sparsity of model weights (Fig 2). Therefore, the hyperparameter *β*_*v*_ served to tune the model capacity via weight sparseness for data prediction.

### System identification incorporates model uncertainty to predict neural responses

**Fig 3:**
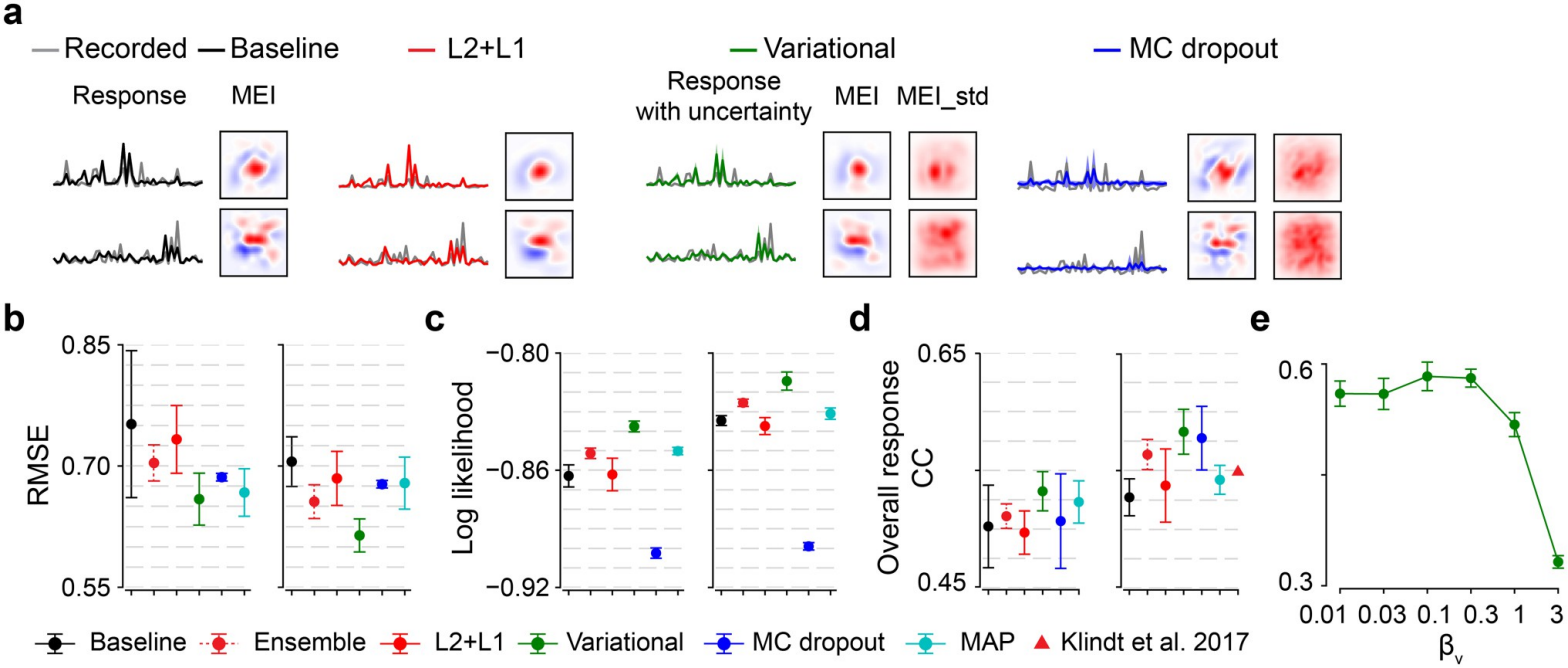
Neural prediction with weight uncertainty. **(a)** Mean recorded responses (gray) and predictive responses to natural stimuli(black, baseline; red, L2+L1; green, variational one with *β*_*v*_ = 0.1; blue, MC dropout with dropout rate 70%; shaded green and blue representing standard deviation for the variational and the dropout methods, respectively), estimated MEIs, as well as standard deviation of MEI (MEI_std; only for two probabilistic models), for two exemplary neurons. MEI and MEI_std use different color scales with red and blue indicating positive and negative values, respectively. Note that MEI has much larger absolute values than MEI_std. **(b)** Predictive performance (RMSE) based on test data with different amounts of training data (left, 50% of training data, *p* = 0.004 for variational vs. baseline, *p* = 0.0024 for variational vs. ensemble, *p* = 0.0006 for variational vs. L2+L1, *p* = 0.0186 for variational vs. MC dropout, *p* = 0.549 for variational vs. MAP, two-sided permutation test with n = 10,000 repeats; right, 100% of data, *p* = 0.0001 for variational vs. baseline, *p* < 0.0001 for variational vs. ensemble, *p* = 0.0001 for variational vs. L2+L1, *p* < 0.0001 for variational vs. MC dropout, *p* = 0.0001 for variational vs. MAP) for 6 models (red dash, ensemble; cyan, MAP; 10 seeds per model). **(c)** Same with (b), but using log likelihood to evaluate models (left, *p* < 0.0001 for variational vs. baseline, *p* < 0.0001 for variational vs. ensemble, *p* < 0.0001 for variational vs. L2+L1, *p* < 0.0001 for variational vs. MC dropout, *p* = 0.0001 for variational vs. MAP; right, *p* = 0.0001 for variational vs. baseline, *p* = 0.0001 for variational vs. ensemble, *p* = 0.0001 for variational vs. L2+L1, *p* = 0.0001 for variational vs. MC dropout, *p* = 0.0001 for variational vs. MAP). **(d)** Same with (b), but using CC to evaluate models (left, *p* = 0.028 for variational vs. baseline, *p* = 0.0043 for variational vs. ensemble, *p* = 0.0009 for variational vs. L2+L1, *p* = 0.082 for variational vs. MC dropout, *p* = 0.2526 for variational vs. MAP; right, *p* = 0.0001 for variational vs. baseline, *p* = 0.013 for variational vs. ensemble, *p* = 0.0007 for variational vs. L2+L1, *p* = 0.6159 for variational vs. MC dropout, *p* = 0.0001 for variational vs. MAP), with another model used by [13] (red triangle). **(e)** Predictive model performance (CC) for different *β*_*v*_ values. Error bars in (b)—(e) represent standard deviation of n = 10 random seeds for each model.

We trained the six models on the dataset 1 (Fig 3a) and tuned their respective hyperparameters using validation data. For the variational model, we found the one with *β*_*v*_ = 0.1 had best predictive performance with a sharp decrease when increasing *β*_*v*_ till 1.0 or 3.0 (S1 Fig). We also observed that at training stage, the variational model presented a more stable performance on validation data compared to the baseline CNN, confirming the regularization effect of prior to prevent overfitting.

Next, we selected the hyperparameters achieving the best performance on validation data for each model. To examine the feature properties learned by these models, we estimated the MEIs of recorded neurons and found that these models yielded antagonistic center-surround and Gabor filters in a local region, reminiscent of neural representations in early visual processing ([1, 3]; Fig 3a and S2). To compare the performance of neural prediction, we then evaluated all models using test data. For a probabilistic model, we ran model predictions for 100 sampling times and computed the mean and the standard deviation of neuronal responses.

We used a significance level of 0.01 after applying a Bonferroni correction. When using RMSE as a metric and 50% of the training data (Fig 3b), we observed that the variational model had similar prediction accuracy with the MAP and MC dropout, and outperformed other methods. When using RMSE and full data, our Bayesian one had higher predictive performance than others. When using log likelihood (Fig 3c), our Bayesian method had significantly higher prediction accuracy than others for both training data sizes. When using linear correlation and half of the data (Fig 3d), the variational one had similar prediction accuracy with MC dropout, the baseline, and MAP, but outperformed the L2+L1 and the ensemble. When using CC as a metric and full training data, our method had similar prediction performance with MC dropout and the ensemble, but outperformed other test models and Klindt’s model [13]. In summary, our results suggested that the variational method had better/comparable predictive performance compared to other models.

We also reanalyzed the influence of *β*_*v*_ on prediction using linear correlation with test data (Fig 3e). Similar to the case with validation data, we noticed a rather steady predictive performance with increasing *β*_*v*_ until a sudden drop at *β*_*v*_ = 1.0 or 3.0, implying that a large Lagrangian multiplier imposing excessive sparsity on weights yields model underfitting. Note that we got best predictive performance when using *β*_*v*_ < 1.0, which is referred to as the cold posterior effect and is consistent with previous results [35–37, 50].

Together, the superior/equivalent performance of our variational approach suggests that incorporating weight uncertainty is beneficial for predicting neural responses.

### Probabilistic models learn variance of neural transfer functions

**Fig 4:**
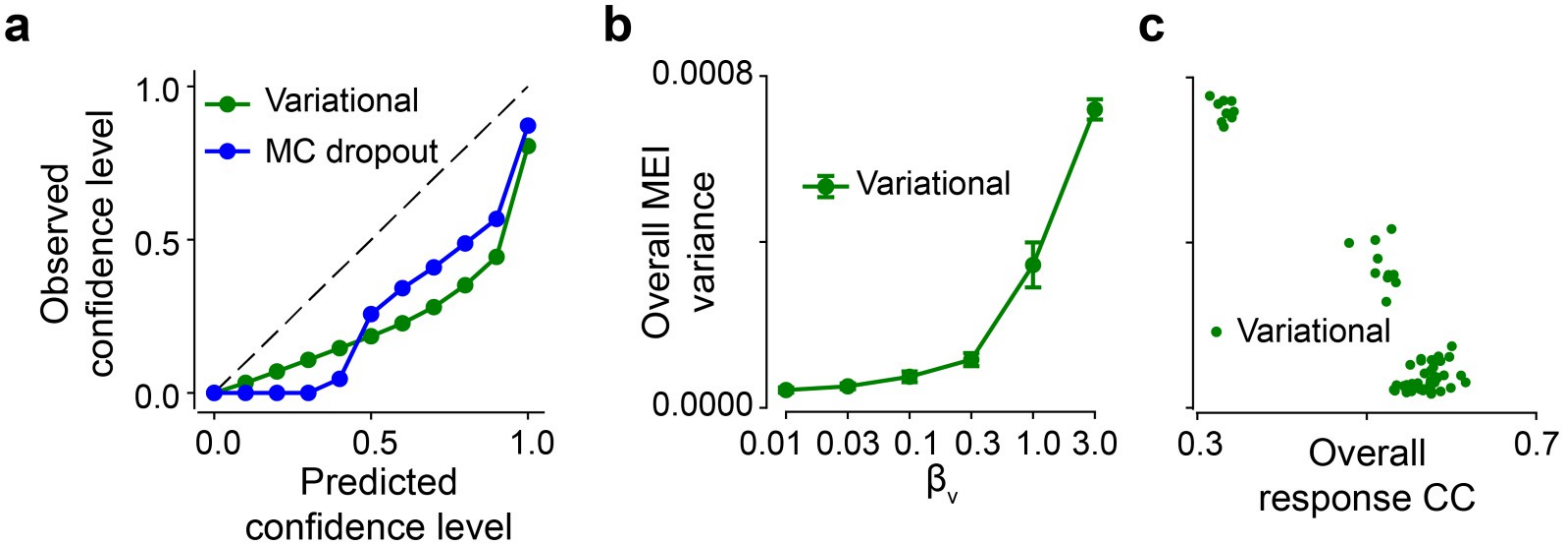
Neural transfer functions with variability. **(a)** Calibration analysis for the variational model and the MC dropout model. The dashed line indicates a perfect calibration curve. **(b)** Overall MEI variance for different *β*_*v*_ values (10 seeds per model). **(c)** Scatter plot of overall response CC and overall MEI variance for 6 *β*_*v*_ values and 10 seeds (each dot representing one model at each *β*_*v*_ and each seed). Error bars in (b) represent standard deviation of n = 10 random seeds for each model.

Probabilistic models predict neuronal responses to test stimuli with uncertainty. We first assessed such uncertainty by conducting standard calibration analysis (Fig 4a). In this way, we can address questions such as whether the predicted 90% credible interval contains the recorded responses 90% of the time. We did calibration analysis on the test data for credible intervals from 0% to 100%, and found that the predicted confidence was higher than the observed confidence for both the variational and MC dropout models. This suggested that both models were overconfident on their predictions, which might be caused by inappropriate prior, suboptimal likelihood function, etc.

Next, we investigated the relationship between predictive performance and response uncertainty using test data (S3 Fig). We did not observe a significant correlation for the variational model (*CC* = 0.24, *p* = 0.05), the MC dropout model (*CC* = 0.18, *p* = 0.07) and the ensemble model (*CC* = 0.19, *p* = 0.06), suggesting no relation between predictive performance and response uncertainty at neuronal level. This might be related to the differential response variability driven by distinct stimuli [51, 52]. Therefore, our results suggest no exact linear correlation between the response uncertainty and predictive performance.

Furthermore, we tested whether the variability of the learned transfer function was related to the predictive performance. At model level, we found a sudden increase of overall MEI variance at *β*_*v*_ = 1.0 or 3.0 (Fig 4b), where an abrupt drop of model performance was present (cf. Fig 3e). This opposite change between MEI variability and predictive performance was confirmed by the negative correlations between overall MEI variance and overall response CC (*CC* = −0.95, *p* < 0.0001; Fig 4c), suggesting a model with higher predictive performance have higher confidence on the learned transfer function. At neuronal level (S3 Fig), we computed the partial correlation between response CC and MEI variance by removing the effect from the mean firing rate. We observed significant correlation for the ensemble model (*CC* = −0.63, *p* < 0.0001), but not for the variational model (*CC* = −0.17, *p* = 0.09) and the MC dropout model (*CC* = −0.07, *p* = 0.50). The inconsistency between these three models suggest no exact linear relationship between prediction accuracy and MEI uncertainty at neuronal level.

In summary, these results demonstrate that, at model level, a probabilistic model with smaller uncertainty on the learned stimulus-response function yields higher predictive performance.

### Variational model features high data efficiency on neural prediction

Here we applied our method on the second dataset shared by Franke and colleagues ([21]). After hyperparameter tuning, we selected *β*_*v*_ = 0.3 for the variational network and evaluated the five models on test data.

**Fig 5:**
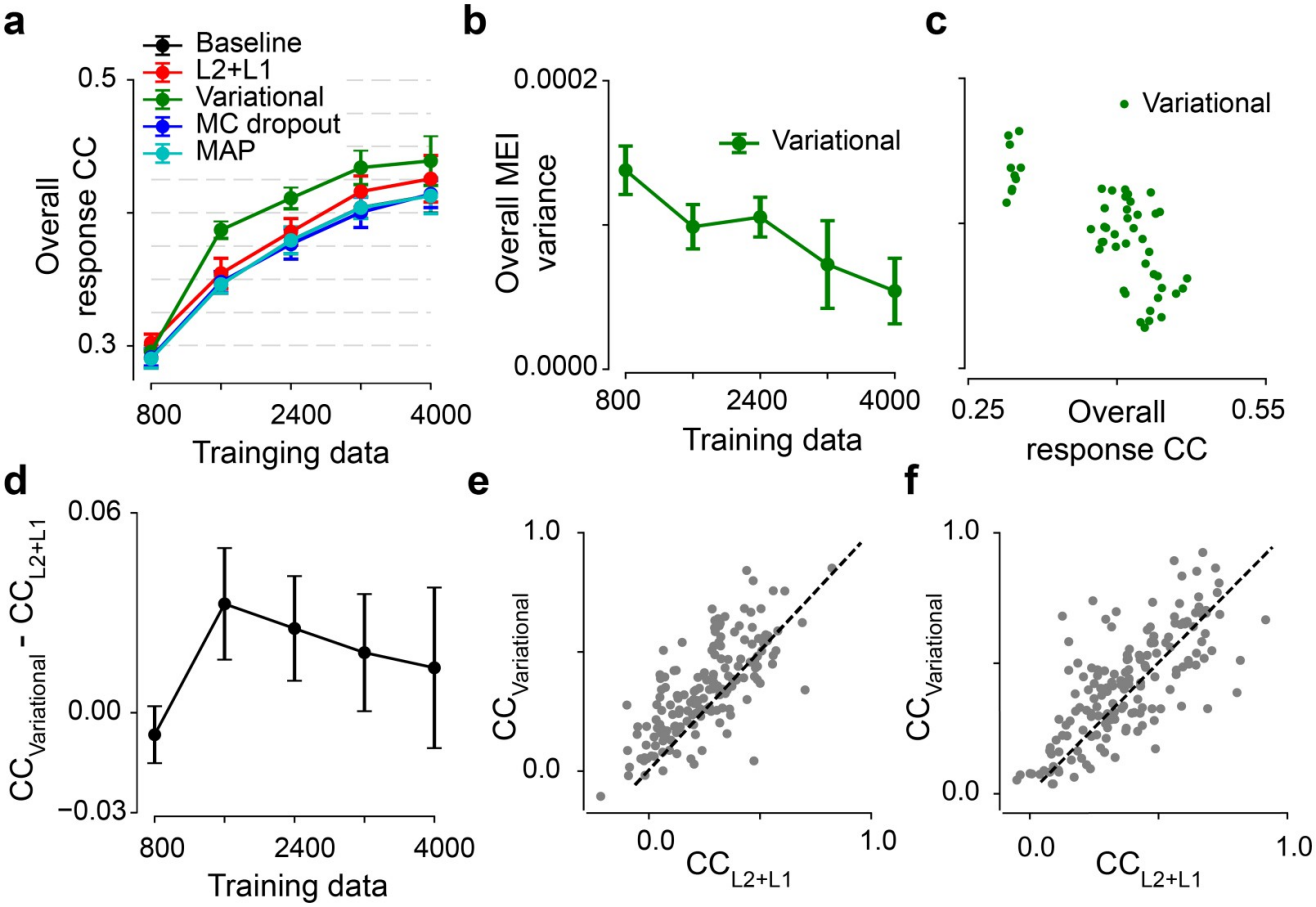
Variational models on the second dataset. **(a)** Model performance based on test data of the second dataset with different amounts of training data for five models (n = 10 random seeds per model). *p* = 0.0238 for variational vs. L2+L1 at 20% of training data, *p* < 0.0001 at 40%, *p* = 0.0001 at 60%, *p* = 0.0042 at 80%, *p* = 0.1096 at 100%. **(b)** Overall MEI variance for different amounts of training data for variational models (10 seeds per model). **(c)** Scatter plot for overall response CC and overall MEI variance for different amounts of training data and at 10 seeds. Each dot representing one model. **(d)** Performance difference between the variational and the L2+L1 models. **(e)** Scatter plot of model predictions for the variational model and the L2+L1 model at one random seed when using 40% training data. Each dot representing one neuron. **(f)** Like (e) but using 100% training data. Error bars in (a), (b) and (d) represent standard deviation of n = 10 random seeds for each model.

We observed that our Bayesian method had better/comparable prediction accuracy compared to other models when using linear correlation, RMSE and log likelihood as evaluation metrics (Fig 5a and S4). We then examined the relationship between the uncertainty of the learned stimulus-response function and the predictive performance measured with CC at model level. We expect that, with more data used for training, the model yields better prediction along with smaller variance for the learned MEIs. We focused on the variational method. Indeed, when more training data was used, the predictive model performance increased (Fig 5a) while the overall MEI variance decreased (Fig 5b), with a negative correlation between them at model level (*CC* = −0.73, *p* < 0.0001; Fig 5c). Note that we did not observe a steady decrease of the overall response variance (S5 Fig).

Next, we investigated whether the performance difference between the variational and the L2+L1 model was sensitive to the training data size (Fig 5d). We observed that the variational method had higher correlations except for the case of extremely little data (20%). The difference peaked at 40% with an increase of 9% (*p* < 0.0001, two-sided permutation test with n = 10,000 repeats) and gradually decreased with more training data, indicating the benefit of variational inference for system identification. We note that such improvement in model performance might vanish in the scenario of unlimited data. We also compared the predictive performance on individual neurons at one random seed and observed that, the Bayesian model outperformed the L2+L1 one for the condition of 40% of training data (*p* < 0.0001; Fig 5e) and two methods had comparable performance for the condition of full data (*p* = 0.0927; Fig 5f).

Together, compared to a traditional method, our Bayesian approach with weight uncertainty yielded higher predictive performance with a higher data efficiency.

### Variational model yields stimuli driving high neuronal activation

**Fig 6:**
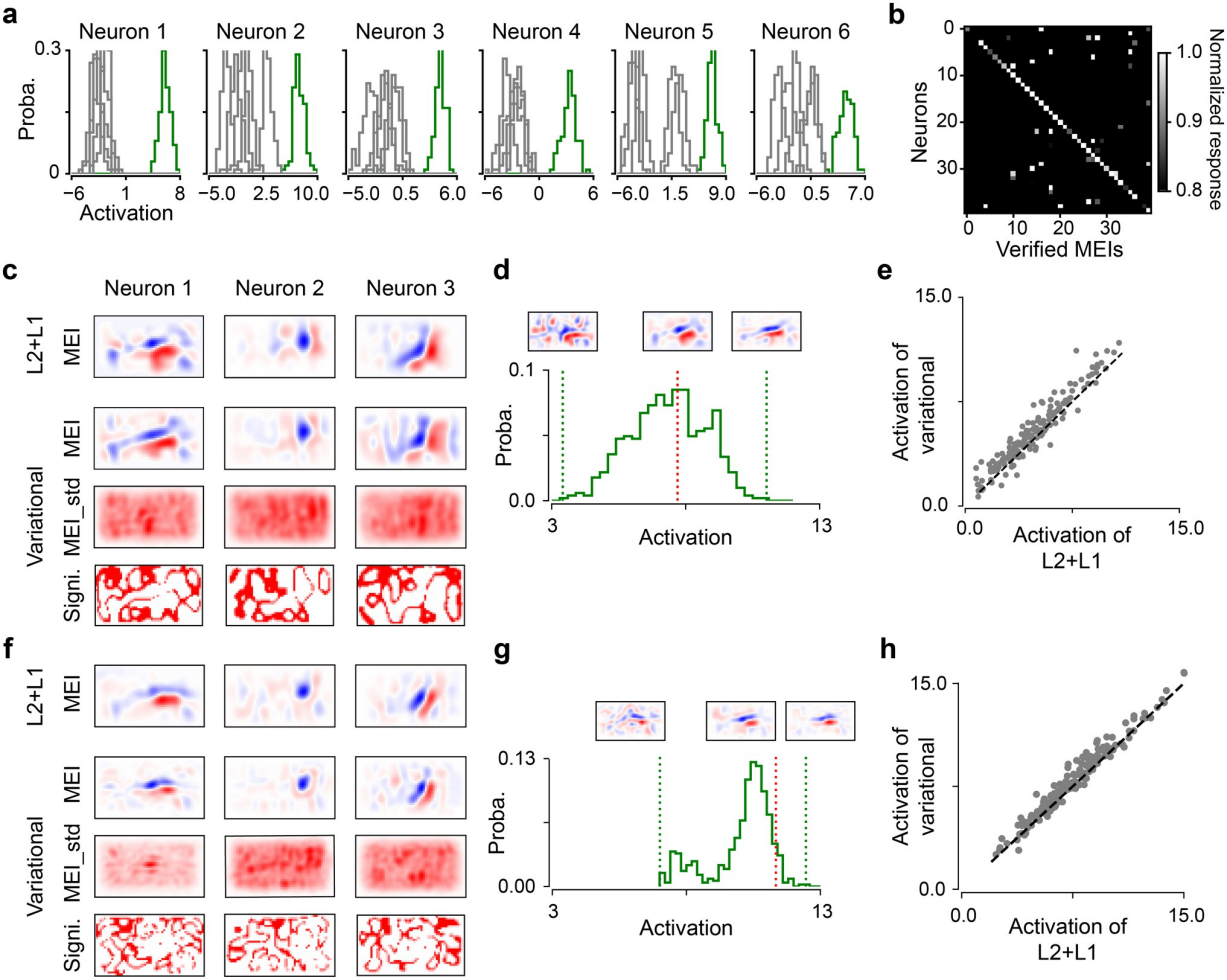
*In silico* experiments of neuronal activity with derived MEIs. **(a)** Activation distributions of 6 exemplary neurons driven by the 6 verified MEIs (green and gray representing the respective MEI and remaining MEIs, respectively). **(b)** Response matrix of each neuron activated by the verified MEIs of all neurons. Scaling was applied to each row to ensure that the maximum of the responses to all stimuli is equal to one. **(c)** Estimated MEIs for L2+L1 (first row) and variational (second row) models, MEI_std (third row), as well as significance map (fourth row; white, *p* < 0.01, one-sample two-sided permutation test against zero for 10,000 repeats), for three exemplary neurons when using 40% of training data. MEI and MEI_std in the UV channel, with different color scales. Note that MEI has much larger absolute values than MEI_std. **(d)** 1D histogram of neuronal activity driven by the generated MEIs from the variational model for Neuron 1 when using 40% of training data. Insets: example MEIs with corresponding activation indicated by dotted lines (red, maximum of L2+L1; green, variational). **(e)** Scatter plot of activation driven by MEIs yielded from variational (using the weight mean *μ*) and L2+L1 models at one random seed when using 40% of training data. Each dot representing one cell. **(f,g,h)** Same with (c), (d) and (e), but using 100% of training data.

Bayesian methods with full posterior provide an infinite ensemble of models for computing MEIs and allow to perform statistical tests for the derived features. Before using the variational model to yield MEIs, we evaluated the effectiveness of our method. We fed the verified MEIs from [21] into the model trained by full data and estimated the neuronal activation driven by distinct stimuli. We assumed that, if a model was well trained, each verified MEI would drive higher activity for the corresponding neuron compared to other stimuli. Firstly, we selected randomly 6 neurons with their MEIs and got 6 activation distributions by sampling the model for 100 times for each neuron (Fig 6a; S7 Fig). We observed that each neuron was activated most by its respective MEI. Furthermore, we used the neurons (n = 40) with available verified MEIs and prediction correlation values exceeding 0.5, and fed these preferred stimuli into our trained model. We observed that 92.5% of neurons were elicited by the respective MEI with more than 80% of highest activity (Fig 6b), suggesting that our Bayesian method works effectively well.

To further assess which method generates the more exciting stimuli for each cell, we conducted *in silico* experiments using a held-out L2+L1 model trained by full data as a digital testbed. We used a CNN model with regularization instead of other models as previous studies have demonstrated its feasibility on yielding cells’ preferred stimuli [20–23]. We fed the MEIs generated by other models to the testbed and compared the neural activity. Specifically, we tested the model using variational inference and the one using L2 and L1 regularization with 40% and 100% of training data.

We found that these learned filters resembled neural features in the early visual system [1, 3] and localized more in the visual field with more training data (Fig 6c and 6f; S8 Fig). Like for the first dataset (Fig 3a), MEI_std was not uniform across visual space, e.g., some presented Gaussian or bar shapes. Additionally, we examined whether the posterior of each pixel differs significantly from zero for the 100 sampled MEIs and found that the significance map may indicate zero-crossings in visual representations. For an example neuron, we measured the responses for all the 506 MEIs yielded from five variational models, and observed that these stimuli drove this neuron with quite different activity, with the maximum response larger than the maximum one yielded (from 6 MEIs) by the traditional models (Fig 6d and 6g). With more training data, the activation distribution shifted towards higher mean with smaller variance. Additionally, we compared the activation on individual neurons for two methods (Fig 6e and 6h), and observed that the Bayesian approach yielded significantly higher responses for the condition using 40% (*p* = 0.0473, two-sided permutation test with n = 10,000 repeats) of training data and comparable responses for the condition using full data (*p* = 0.2114).

In summary, our variational model allowed statistical test for the derived response functions and yielded the stimuli driving neurons better than traditional methods in the limited data regime, suggesting that weight uncertainty benefits the learning of neural representations.

## Discussion

We presented a Bayesian approach for identification of neural properties by incorporating model uncertainty through learning the distribution of model weights, aiming to estimate neural features with credible intervals. Our empirical results on different datasets show that the variational method had higher or comparable predictive performance, especially in the limited data regime, compared to methods using dropout or traditional methods learning point estimates of model parameters. Moreover, by sampling from posterior distribution of model weights, our approach enabled to provide credible intervals and test statistics for the learned MEIs, avoiding the idiosyncrasy of a single model. Finally, *in silico* experiments show that the variational model yielded the MEIs driving neurons with higher activity compared to the traditional model when limited data were used for training. This suggests that model uncertainty contributes to learning neural transfer functions with a high data efficiency.

### Variational models with cold posterior effects

Our Bayesian method had best prediction accuracy when using log likelihood as an evaluation metric. The results with three metrics demonstrated that the variational model had higher/comparable predictive performance compared to others. We noticed that the improvement was marginal for many conditions, primarily occuring in a limited data regime. Such improvement was sensitive to the data size and might disappear with the increase of training data. However, high data efficiency is of great importance given the high cost of data collection and the limited recording time in neuroscience. A common scenario is that non-linear response functions, with a high-dimensional stimulus space, are learned from limited amounts of recorded data.

Compared to a standard objective function, we used *β*_*v*_ to adjust the weighting between the prior and the likelihood and achieved the best predictive performance with *β*_*v*_ < 1.0. Wenzel et al. [35] did many hypothesis tests to study the origin of this cold posterior effect, including minibatch noise, bias-variance tradeoff, dirty likelihood, prior variance, training set size, model capacity and initialization. They showed that the inappropriate prior was related to the predictive performance improvement for *β*_*v*_ < 1.0 and claimed that this was probably not the only cause. Therefore, we should be more careful when interpreting the inferred posterior. In fact, we did observe that the variational method and the MC dropout method underestimated the uncertainty of model parameters, suggesting future work on calibrating these models [53, 54]. Additionally, such calibration analysis could be used in future studies to evaluate model reliability, compare model assumptions and guide model selections. Furthermore, it might be interesting to use proper scoring rules, e.g., Continuous Ranked Probability Score, to evaluate the accuracy of probabilistic predictions for the variational method [55, 56].

### Relation to trial-to-trial variability

Neural information process is probabilistic, i.e., neurons respond with trial-to-trial fluctuations to a repeated presentation of a stimulus [57, 58]. Response variability is found across neural systems, originating from diverse factors, such as synapse variation, channel noise, brain state, and attention [52, 59–63]. Additionally, the variability between populations of neurons are correlated. In a simplified case, a pair of neurons may present correlations for the single-trial responses, i.e., pairwise noise correlation, which also contributes to neural coding ([64]; reviewed in [65–68]). Such response variability is inherent in neural data itself and is a kind of aleatoric but not epistemic uncertainty. We note that the standard deviations of the estimated MEIs from our models decreased with the increasing amounts of training data, suggesting that the variability of the sampled predicted responses may not be related to the response uncertainty in biological neurons or our models may predict a mix of both uncertainties (S6 Fig). Additionally, the uncertainty in *q*(**w**|*θ*) should be primarily related to the epistemic uncertainty, it could converge to zero in the unlimited-data regime. Still, it may be related to aleatoric uncertainty too, as we may use suboptimal loss functions and model architectures, both of which may also reflect a model’s inability to capture the inherent noise in data.

### Necessity of uncertainty quantification for yielded preferred stimuli

Though DNN approaches have demonstrated remarkable power in predicting neural responses to diverse stimuli and generating novel hypothesis about neuronal features, they require significant amounts of stimulus-response pair data for the training. Besides the epistemic uncertainty introduced by limited data, such hypothesis also entails further closed-loop animal experiments to verify the derived properties, which consumes much experimental time [20, 23]. Still, it is impossible to confirm the yielded preferred stimuli for all neurons across the high-dimensional stimulus space with experiments. Practically, only a subset of cells are selected for verification. Probabilistic MEIs allow us to pinpoint those neurons with interesting but subtle/uncertain derived features. Therefore, it is critical to quantify the uncertainty of the yielded representations for all recorded neurons [69, 70]. Additionally, the credible interval of the derived features offers an opportunity to generate an ensemble of infinite preferred stimuli. An interesting study would be to compare the neuronal activity driven by these similar MEIs in animal experiments, which may allow to test the robustness of the biological system.

We note that, even for a model using point estimate of parameters (such as L2+L1), it may yield different preferred stimuli by initializing the MEI generation randomly. Yet, this uncertainty depends on the starting points of non-convex optimizations, rather than the training data. Empirically, we found that such variance was quite stable when using L2+L1 models with different amounts of training data and was also much smaller than the MEI variance we computed (cf. Fig 5b). Therefore, the measure of epistemic uncertainty calls for a Bayesian framework or an ensemble of many models.

Bayesian models, unlike traditional ‘point-estimation’ approaches, offer the advantage of quantifying the uncertainty of inferred neural features. Nevertheless, in seeking to capture the full distribution of weights, they entail increased complexity including model parameters and hyperparameters, which in turn necessitate higher computational demands and may pose challenges for interpretation.

### Future work & general impact

Incorporating uncertainty to DNNs have flourished in recent years [reviewed in 53, 54], including Bayesian methods which specify a prior distribution for network weights and approximate the full posterior given the training data using different tricks such as variational inference [32, 71], Laplace approximation [72, 73] and expectation propagation [74]. Non-Bayesian methods include applying MC dropout in the network [44] or training an ensemble of models that are initialized by different seeds [75]. In the future, our variational approach could be extended by more advanced methods such as incorporating correlated model parameters [71] or jointly considering model and parameter uncertainty [76]. Additionally, other uncertainty representation methods such as Stochastic Weight Averaging-Gaussian [77] are interesting and promising directions, especially for large-scale model architectures and datasets. While these methods are powerful to predict uncertainty, it would be interesting to investigate biologically inspired methods such as adding noise to network parameters/activation in the future.

We used a Poisson loss function to train our model, which may not be optimal considering that neuronal responses may present non-exact Poisson behavior [51]. In this case, our model may capture only a part of trial-to-trial variability in neural data. Such response fluctuation depends on many conditions, including biochemical process, internal brain states and engaged behavioral tasks [51, 52, 59, 60]. These factors have been described by a low-dimensional latent state models [52, 78, 79]. Therefore, a potential extension of our method could be a variational network incorporated with latent state variables.

Bayesian frameworks could potentially advance neural prediction in multiple ways. One promising direction is Bayesian model comparison. It enables us to select model hyperparameters and network architectures with promising out-of-distribution detection performance by computing marginal likelihood. However, it is challenging in deep neural networks and requires additional estimation methods such as Gauss-Newton approximations to the Hessian [80–82]. A starting point could be to try stochastic variational inference which estimates both model and weight uncertainty [76]. Another interesting research is to estimate the amount of data for achieving performance saturation for specific animal-electrode noise-recording method combinations. This may involve leveraging techniques such as Bayesian optimization [83, 84] and Bayesian experimental design [85, 86]. However, it may pose a considerable challenge. Notably, neurons at different brain regions may feature different levels of nonlinear processing. As a result, cells at higher visual areas probably demand more training data compared to at the retina. Additionally, it is important to calibrate a probabilistic model, which could overestimate/underestimate the uncertainty of the posterior. Furthermore, trial-to-trial variability inherent in neural data varies across brain regions [51], potentially influencing predictive performance as well. Considering our model may not capture such aleatoric uncertainty well, alternative probabilistic methods may be demanded. A promising start may be to use Bayesian experimental design to actively select the informative stimuli during closed-loop experiments to fit response functions and distinguish between models [24].

Our *in silico* experiments indicate that the stimuli generated by the variational model driving higher neuronal activation than the CNN with regularization, which requires future animal experiments to test. Additionally, we noticed that the MEI_std was not uniform in the visual field for each neuron and its location was not overlaid with the central MEI, for example, it seems to sit on the surround of the corresponding MEI. It would be interesting to examine and quantify the MEI uncertainty in regard of visual space, which might be related to contextual sensory processing [87–89].

More generally, why do we care about the uncertainty of the estimated neural representations? Even with closed-loop experiments, it is impossible for us to test all potential (preferred) inputs for the recorded neurons [20, 21, 23]. Therefore, we always expect to have a confidence interval for the test statistics. Besides, a Bayesian model offers a manner to generate many stimulus candidates by sampling for stimulating neural systems, which may offer new insights for understanding the biological computation.

## Supporting information

S1 Appendix Additional model details. (PDF)

S1 Fig Neural prediction for first dataset. **(a,b)** Predictive performance (correlation coefficient, CC) based on validation data during training for variational models (*β*_*v*_ = 0.1) with different prior distributions. All layers adopted the same *σ*_2_ = exp(−6) with different *π* and *σ*_1_ values (a), or with the same parameters of prior distribution (b). We picked *π* = 0.5, *σ*_1_ = 1, *σ*_2_ = exp(−6) for subsequent model training. **(c)** Predictive performance based on validation data during model training for different numbers of Monte Carlo sampling. We picked number = 1 or 2 to save training time. **(d)** Model performance based on validation data during training for the baseline and variatonal models with different *β*_*v*_ values. **(e)** Overall variance of predicted responses to test stimuli for different *β*_*v*_ values. **(f)** Histogram of response variance (top) and MEI (RF) variance (bottom) for the variational and the MC dropout models. Dotted line represents the mean of histogram. **(g)** Model performance (left) based on test data and RF overall variance (right) for two probabilistic models with different amounts of training data. Error bars in (e) and (g) represent standard deviation of n = 10 random seeds for each model.
(TIF)

S2 Fig Estimated MEIs for first dataset. **(a,b)** MEIs of 30 exemplary neurons for the first dataset generated by the L2+L1 model (a) and the variational one (b).
(TIF)

S3 Fig Uncertainty analysis for first dataset. **(a)** Scatter plot of response CC and response variance for variational model at one random seed (each dot representing one neuron; *CC* = 0.24, *p* = 0.05). **(b)** Scatter plot of response CC and MEI variance for variational model at one random seed (each dot representing one neuron; *CC* = −0.37, *p* = 0.0001). **(c)** Scatter plot of mean firing rate and MEI variance for variational model at one random seed (each dot representing one neuron; *CC* = −0.47, *p* < 0.0001). **(d)** Same with (a), but for MC dropout model (*CC* = 0.18, *p* = 0.07). **(e)** Same with (b), but for MC dropout model (*CC* = −0.23, *p* = 0.02). **(f)** Same with (c), but for MC dropout model (*CC* = −0.33, *p* = 0.006). **(g)** Same with (a), but for ensemble model (*CC* = 0.19, *p* = 0.06). **(h)** Same with (b), but for ensemble model (*CC* = −0.34, *p* = 0.0004). **(i)** Same with (c), but for ensemble model (*CC* = −0.35, *p* = 0.0003).
(TIF)

S4 Fig Model evaluation using RMSE and log likelihood for second dataset. **(a,b)** Like Fig 5a but using RMSE (a) and log likelihood (b) to compare models. When using RMSE, we found our variational method had equivalent prediction accuracy to the MC dropout model in the condition of full data (p = 0.1709), and the Bayesian one outperformed the MC dropout one in conditions of less data (*p* = 0.0001 at 20% of data, *p* < 0.0001 at 40%, *p* = 0.0001 at 60%, *p* = 0.0021 at 80%). When using log likelihood, the variational model had significantly higher predictive performance than the MC dropout method (*p* = 0.0002 at 20%, *p* < 0.0001 at 40%, *p* < 0.0001 at 60%, *p* < 0.0001 at 80%, *p* < 0.0001 at 100%).
(TIF)

S5 Fig Neural prediction for second dataset. **(a)** Model performance based on validation data during training for the baseline and the variational models with different *β*_*v*_ values. **(b,c)** Scatter plot of response CC and MEI (RF) variance for MC dropout (b) and variational (c) models for 10 seeds (*CC* = −0.25, *p* = 0.001 and *CC* = −0.34, *p* < 0.0001 for dropout and variational one, each dot representing one neuron at one random seed). **(d)** Predictive performance, overall RF variance and overall response variance for variational models with different *β*_*v*_ values. **(c)** Predictive performance based on validation data during model training for different numbers of Monte Carlo sampling. We picked number = 1 or 2 to save training time. **(d)** Model performance based on validation data during training for the baseline and the variational ones with different *β*_*v*_ values. **(e)** Scatter plot for overall response CC and overall RF variance for the variational methods with different *β*_*v*_ values (d) and at 10 seeds (*CC* = −0.82, *p* < 0.0001). Each dot represents one model. **(f)** Overall response variance for different amounts of training data for the variational models (10 seeds per model). **(g)** Scatter plot for overall response CC and overall RF variance for the dropout model with different amounts of training data and at 10 seeds (*CC* = −0.17, *p* = 0.24). Each dot represents one model. Error bars in (d) and (f) represent standard deviation of n = 10 random seeds for each model.
(TIF)

S6 Fig Variance of predicted vs. recorded responses for second dataset. Using the trained models, we tested whether the variance of predicted responses was related to the variance of recorded responses for each neuron. We first estimated the predicted response variance to a stimulus. For the L2+L1 model, as the mean of neural responses is proportional to the variance, we used the model output (a single predicted value) as a substitute. For the variational one, we either used the mean of predicted responses (multiple sampling times) as a substitute or calculated the response variance explicitly. **(a)** Scatter plot (axes in log scale) of predicted response variance (using response mean as a substitute) and recorded response variance for one neuron for a variational model. Each dot representing one stimulus. **(b)** Distribution of correlations between recorded and predicted response variance for all neurons for the L2+L1, variational-mean (using response mean as a substitute) and variational-variance (calculating response variance), at one random seed. Horizontal lines representing distribution means. **(c)** Mean correlations between two response variances (10 seeds per model). Note that variational-variance had lower correlation than the L2+L1. Error bars represent standard deviation of n = 10 random seeds for each model. We computed the correlation using the predicted and recorded response variances of the test stimuli for each neuron (*CC* = 0.34, *p* = 0.002, Spearman correlation for an exemplary neuron; a). We found that the variational one using response mean as a substitute of variance had a slightly higher mean correlation across neurons compared to the L2+L1 (*p* = 0.0368, two-sided permutation tests on 10 random seeds for 10,000 times; b,c).
(TIF)

S7 Fig Neural activation test with verified MEIs for second dataset. Instead of performing closed-loop experiments to examine the effectiveness of our veriational model, we used the verified MEIs from previous study to compare the neuronal activities driven by different stimuli [21]. We plotted 1D histograms of activation of 6 exemplary cells (from left to right) driven by the verified MEIs (from top to bottom). We used green color instead of gray to highlight the highest neuronal activation on the diagonal (driven by the respective verified MEI).
(TIF)

S8 Fig Estimated MEIs for second dataset when using full training data. **(a,b)** MEIs in the UV channel of 30 exemplary neurons generated by L2+L1 model (a) and variational one (b).
(TIF)

## References

[1] Hubel DH, Wiesel TN. Receptive fields of single neurones in the cat’s striate cortex. The Journal of physiology. 1959;148(3):574. doi: 10.1113/jphysiol.1959.sp006308 14403679 PMC1363130

[2] Wu MCK, David SV, Gallant JL. Complete functional characterization of sensory neurons by system identification. Annu Rev Neurosci. 2006;29:477–505. doi: 10.1146/annurev.neuro.29.051605.113024 16776594

[3] Chichilnisky E. A simple white noise analysis of neuronal light responses. Network: Computation in Neural Systems. 2001;12(2):199–213. doi: 10.1080/713663221 11405422

[4] Pillow JW, Shlens J, Paninski L, Sher A, Litke AM, Chichilnisky E, et al. Spatio-temporal correlations and visual signalling in a complete neuronal population. Nature. 2008;454(7207):995–999. doi: 10.1038/nature07140 18650810 PMC2684455

[5] Huang Z, Ran Y, Oesterle J, Euler T, Berens P. Estimating smooth and sparse neural receptive fields with a flexible spline basis. arXiv preprint arXiv:210807537. 2021;.

[6] Karamanlis D, Gollisch T. Nonlinear spatial integration underlies the diversity of retinal ganglion cell responses to natural images. Journal of Neuroscience. 2021;41(15):3479–3498. doi: 10.1523/JNEUROSCI.3075-20.2021 33664129 PMC8051676

[7] Rust NC, Movshon JA. In praise of artifice. Nature neuroscience. 2005;8(12):1647–1650. doi: 10.1038/nn1606 16306892

[8] Qiu Y, Zhao Z, Klindt D, Kautzky M, Szatko KP, Schaeffel F, et al. Natural environment statistics in the upper and lower visual field are reflected in mouse retinal specializations. Current Biology. 2021;31(15):3233–3247. doi: 10.1016/j.cub.2021.05.017 34107304

[9] Qiu Y, Klindt DA, Szatko KP, Gonschorek D, Hoefling L, Schubert T, et al. Efficient coding of natural scenes improves neural system identification. PLOS Computational Biology. 2023;19(4):e1011037. doi: 10.1371/journal.pcbi.1011037 37093861 PMC10159360

[10] McIntosh L, Maheswaranathan N, Nayebi A, Ganguli S, Baccus S. Deep learning models of the retinal response to natural scenes. In: Advances in neural information processing systems; 2016. p. 1369–1377. 28729779 PMC5515384

[11] Batty E, Merel J, Brackbill N, Heitman A, Sher A, Litke A, et al. Multilayer recurrent network models of primate retinal ganglion cell responses. 2016;.

[12] Antolík J, Hofer SB, Bednar JA, Mrsic-Flogel TD. Model constrained by visual hierarchy improves prediction of neural responses to natural scenes. PLoS computational biology. 2016;12(6):e1004927. doi: 10.1371/journal.pcbi.1004927 27348548 PMC4922657

[13] Klindt D, Ecker AS, Euler T, Bethge M. Neural system identification for large populations separating “what” and “where”. In: Advances in Neural Information Processing Systems; 2017. p. 3506–3516.

[14] Ecker AS, Sinz FH, Froudarakis E, Fahey PG, Cadena SA, Walker EY, et al. A rotation-equivariant convolutional neural network model of primary visual cortex. arXiv preprint arXiv:180910504. 2018;.

[15] Lurz KK, Bashiri M, Willeke K, Jagadish AK, Wang E, Walker EY, et al. Generalization in data-driven models of primary visual cortex. BioRxiv. 2021; p. 2020–10.

[16] Yamins DL, Hong H, Cadieu CF, Solomon EA, Seibert D, DiCarlo JJ. Performance-optimized hierarchical models predict neural responses in higher visual cortex. Proceedings of the National Academy of Sciences. 2014;111(23):8619–8624. doi: 10.1073/pnas.1403112111 24812127 PMC4060707

[17] Güçlü U, van Gerven MA. Deep neural networks reveal a gradient in the complexity of neural representations across the ventral stream. Journal of Neuroscience. 2015;35(27):10005–10014. doi: 10.1523/JNEUROSCI.5023-14.2015 26157000 PMC6605414

[18] Bashivan P, Kar K, DiCarlo JJ. Neural population control via deep image synthesis. Science. 2019;364 (6439). doi: 10.1126/science.aav9436 31048462

[19] Ponce CR, Xiao W, Schade PF, Hartmann TS, Kreiman G, Livingstone MS. Evolving images for visual neurons using a deep generative network reveals coding principles and neuronal preferences. Cell. 2019;177(4):999–1009. doi: 10.1016/j.cell.2019.04.005 31051108 PMC6718199

[20] Walker EY, Sinz FH, Cobos E, Muhammad T, Froudarakis E, Fahey PG, et al. Inception loops discover what excites neurons most using deep predictive models. Nature neuroscience. 2019;22(12):2060–2065. doi: 10.1038/s41593-019-0517-x 31686023

[21] Franke K, Willeke KF, Ponder K, Galdamez M, Muhammad T, Patel S, et al. Behavioral state tunes mouse vision to ethological features through pupil dilation. bioRxiv. 2021;.

[22] Hoefling L, Szatko KP, Behrens C, Qiu Y, Klindt DA, Jessen Z, et al. A chromatic feature detector in the retina signals visual context changes. bioRxiv. 2022;. 10.7554/eLife.86860 PMC11452179 39365730

[23] Tong R, da Silva R, Lin D, Ghosh A, Wilsenach J, Cianfarano E, et al. The feature landscape of visual cortex. bioRxiv. 2023; p. 2023–11.

[24] Goldin MA, Virgili S, Chalk M. Scalable Gaussian process inference of neural responses to natural images. Proceedings of the National Academy of Sciences. 2023;120(34):e2301150120. doi: 10.1073/pnas.2301150120 37579153 PMC10450671

[25] Cotton RJ, Sinz F, Tolias A. Factorized neural processes for neural processes: K-shot prediction of neural responses. Advances in Neural Information Processing Systems. 2020;33:11368–11379.

[26] Gerwinn S, Bethge M, Macke JH, Seeger M. Bayesian inference for spiking neuron models with a sparsity prior. Advances in neural information processing systems. 2007;20.

[27] Gerwinn S, Macke JH, Bethge M. Bayesian inference for generalized linear models for spiking neurons. Frontiers in computational neuroscience. 2010;4:1299. doi: 10.3389/fncom.2010.00012 20577627 PMC2889714

[28] Park IM, Pillow J. Bayesian spike-triggered covariance analysis. Advances in neural information processing systems. 2011;24.

[29] Hinton GE, Van Camp D. Keeping the neural networks simple by minimizing the description length of the weights. In: Proceedings of the sixth annual conference on Computational learning theory; 1993. p. 5–13.

[30] Neal RM, Hinton GE. A view of the EM algorithm that justifies incremental, sparse, and other variants. In: Learning in graphical models. Springer; 1998. p. 355–368.

[31] Jaakkola TS, Jordan MI. Bayesian parameter estimation via variational methods. Statistics and Computing. 2000;10(1):25–37. doi: 10.1023/A:1008932416310

[32] Blundell C, Cornebise J, Kavukcuoglu K, Wierstra D. Weight uncertainty in neural networks. arXiv preprint arXiv:150505424. 2015;.

[33] Higgins I, Matthey L, Pal A, Burgess C, Glorot X, Botvinick M, et al. beta-vae: Learning basic visual concepts with a constrained variational framework. 2016;.

[34] Kuhn HW, Tucker AW. Nonlinear programming. In: Proceedings of 2nd Berkeley Symposium; 1951. p. 481–492.

[35] Wenzel F, Roth K, Veeling BS, Światkowski J, Tran L, Mandt S, et al. How good is the Bayes posterior in deep neural networks really? arXiv preprint arXiv:200202405. 2020;.

[36] Zhang G, Sun S, Duvenaud D, Grosse R. Noisy natural gradient as variational inference. In: International conference on machine learning. PMLR; 2018. p. 5852–5861.

[37] Osawa K, Swaroop S, Khan MEE, Jain A, Eschenhagen R, Turner RE, et al. Practical deep learning with Bayesian principles. Advances in neural information processing systems. 2019;32.

[38] Fortuin V, Garriga-Alonso A, Ober SW, Wenzel F, Rätsch G, Turner RE, et al. Bayesian neural network priors revisited. arXiv preprint arXiv:210206571. 2021;.

[39] Field DJ. What is the goal of sensory coding? Neural computation. 1994;6(4):559–601. doi: 10.1162/neco.1994.6.4.559

[40] Olshausen BA, Field DJ. Emergence of simple-cell receptive field properties by learning a sparse code for natural images. Nature. 1996;381(6583):607–609. doi: 10.1038/381607a0 8637596

[41] Olshausen B, Millman K. Learning sparse codes with a mixture-of-Gaussians prior. Advances in neural information processing systems. 1999;12.

[42] Stevenson IH, Rebesco JM, Hatsopoulos NG, Haga Z, Miller LE, Kording KP. Bayesian inference of functional connectivity and network structure from spikes. IEEE Transactions on Neural Systems and Rehabilitation Engineering. 2008;17(3):203–213. doi: 10.1109/TNSRE.2008.2010471 19273038 PMC3406607

[43] Srivastava N, Hinton G, Krizhevsky A, Sutskever I, Salakhutdinov R. Dropout: a simple way to prevent neural networks from overfitting. The journal of machine learning research. 2014;15(1):1929–1958.

[44] Gal Y, Ghahramani Z. Dropout as a bayesian approximation: Representing model uncertainty in deep learning. In: international conference on machine learning. PMLR; 2016. p. 1050–1059.

[45] Kingma DP, Welling M. Auto-encoding variational bayes. arXiv preprint arXiv:13126114. 2013;.

[46] Erhan D, Bengio Y, Courville A, Vincent P. Visualizing higher-layer features of a deep network. University of Montreal. 2009;1341(3):1.

[47] Alemi AA, Fischer I, Dillon JV, Murphy K. Deep variational information bottleneck. arXiv preprint arXiv:161200410. 2016;.

[48] Tishby N, Pereira FC, Bialek W. The information bottleneck method. arXiv preprint physics/0004057. 2000;.

[49] Burgess CP, Higgins I, Pal A, Matthey L, Watters N, Desjardins G, et al. Understanding disentangling in *β*-VAE. arXiv preprint arXiv:180403599. 2018;.

[50] Ashukha A, Lyzhov A, Molchanov D, Vetrov D. Pitfalls of in-domain uncertainty estimation and ensembling in deep learning. arXiv preprint arXiv:200206470. 2020;.

[51] Goris RL, Movshon JA, Simoncelli EP. Partitioning neuronal variability. Nature neuroscience. 2014;17(6):858–865. doi: 10.1038/nn.3711 24777419 PMC4135707

[52] Ecker AS, Berens P, Cotton RJ, Subramaniyan M, Denfield GH, Cadwell CR, et al. State dependence of noise correlations in macaque primary visual cortex. Neuron. 2014;82(1):235–248. doi: 10.1016/j.neuron.2014.02.006 24698278 PMC3990250

[53] Abdar M, Pourpanah F, Hussain S, Rezazadegan D, Liu L, Ghavamzadeh M, et al. A review of uncertainty quantification in deep learning: Techniques, applications and challenges. Information fusion. 2021;76:243–297. doi: 10.1016/j.inffus.2021.05.008

[54] Gawlikowski J, Tassi CRN, Ali M, Lee J, Humt M, Feng J, et al. A survey of uncertainty in deep neural networks. Artificial Intelligence Review. 2023;56(Suppl 1):1513–1589. doi: 10.1007/s10462-023-10562-9

[55] Matheson JE, Winkler RL. Scoring rules for continuous probability distributions. Management science. 1976;22(10):1087–1096. doi: 10.1287/mnsc.22.10.1087

[56] Gneiting T, Raftery AE. Strictly proper scoring rules, prediction, and estimation. Journal of the American statistical Association. 2007;102(477):359–378. doi: 10.1198/016214506000001437

[57] Perkel DH, Gerstein GL, Moore GP. Neuronal spike trains and stochastic point processes: II. Simultaneous spike trains. Biophysical journal. 1967;7(4):419–440. doi: 10.1016/S0006-3495(67)86597-4 4292792 PMC1368069

[58] Stein RB. Some models of neuronal variability. Biophysical journal. 1967;7(1):37–68. doi: 10.1016/S0006-3495(67)86574-3 19210981 PMC1368056

[59] Faisal AA, Selen LP, Wolpert DM. Noise in the nervous system. Nature reviews neuroscience. 2008;9(4):292–303. doi: 10.1038/nrn2258 18319728 PMC2631351

[60] Mitchell JF, Sundberg KA, Reynolds JH. Spatial attention decorrelates intrinsic activity fluctuations in macaque area V4. Neuron. 2009;63(6):879–888. doi: 10.1016/j.neuron.2009.09.013 19778515 PMC2765230

[61] Cohen MR, Newsome WT. Context-dependent changes in functional circuitry in visual area MT. Neuron. 2008;60(1):162–173. doi: 10.1016/j.neuron.2008.08.007 18940596 PMC2652654

[62] Cohen MR, Maunsell JH. Attention improves performance primarily by reducing interneuronal correlations. Nature neuroscience. 2009;12(12):1594–1600. doi: 10.1038/nn.2439 19915566 PMC2820564

[63] Ecker AS, Berens P, Keliris GA, Bethge M, Logothetis NK, Tolias AS. Decorrelated neuronal firing in cortical microcircuits. science. 2010;327(5965):584–587. doi: 10.1126/science.1179867 20110506

[64] Abbott LF, Dayan P. The effect of correlated variability on the accuracy of a population code. Neural computation. 1999;11(1):91–101. doi: 10.1162/089976699300016827 9950724

[65] Averbeck BB, Latham PE, Pouget A. Neural correlations, population coding and computation. Nature reviews neuroscience. 2006;7(5):358–366. doi: 10.1038/nrn1888 16760916

[66] Kohn A, Coen-Cagli R, Kanitscheider I, Pouget A. Correlations and neuronal population information. Annual review of neuroscience. 2016;39:237. doi: 10.1146/annurev-neuro-070815-013851 27145916 PMC5137197

[67] Doiron B, Litwin-Kumar A, Rosenbaum R, Ocker GK, Josić K. The mechanics of state-dependent neural correlations. Nature neuroscience. 2016;19(3):383–393. doi: 10.1038/nn.4242 26906505 PMC5477791

[68] Da Silveira RA, Rieke F. The geometry of information coding in correlated neural populations. arXiv preprint arXiv:210200772. 2021;. 10.1146/annurev-neuro-120320-082744 33863252

[69] Richards BA, Lillicrap TP, Beaudoin P, Bengio Y, Bogacz R, Christensen A, et al. A deep learning framework for neuroscience. Nature neuroscience. 2019;22(11):1761–1770. doi: 10.1038/s41593-019-0520-2 31659335 PMC7115933

[70] Saxe A, Nelli S, Summerfield C. If deep learning is the answer, what is the question? Nature Reviews Neuroscience. 2021;22(1):55–67. doi: 10.1038/s41583-020-00395-8 33199854

[71] Posch K, Pilz J. Correlated parameters to accurately measure uncertainty in deep neural networks. IEEE Transactions on Neural Networks and Learning Systems. 2020;32(3):1037–1051. doi: 10.1109/TNNLS.2020.2980004 32310784

[72] Mackay DJC. Bayesian methods for adaptive models. California Institute of Technology; 1992.

[73] Ritter H, Botev A, Barber D. A scalable laplace approximation for neural networks. In: 6th International Conference on Learning Representations, ICLR 2018-Conference Track Proceedings. vol. 6. International Conference on Representation Learning; 2018.

[74] Li Y, Hernández-Lobato JM, Turner RE. Stochastic expectation propagation. Advances in neural information processing systems. 2015;28.

[75] Lakshminarayanan B, Pritzel A, Blundell C. Simple and scalable predictive uncertainty estimation using deep ensembles. Advances in neural information processing systems. 2017;30.

[76] Hubin A, Storvik G. Combining model and parameter uncertainty in Bayesian neural networks. arXiv preprint arXiv:190307594. 2019;.

[77] Maddox WJ, Izmailov P, Garipov T, Vetrov DP, Wilson AG. A simple baseline for bayesian uncertainty in deep learning. Advances in neural information processing systems. 2019;32.

[78] Yu BM, Cunningham JP, Santhanam G, Ryu S, Shenoy KV, Sahani M. Gaussian-process factor analysis for low-dimensional single-trial analysis of neural population activity. Advances in neural information processing systems. 2008;21. 10.1152/jn.90941.2008 PMC2712272 19357332

[79] Bashiri M, Walker E, Lurz KK, Jagadish A, Muhammad T, Ding Z, et al. A flow-based latent state generative model of neural population responses to natural images. Advances in Neural Information Processing Systems. 2021;34:15801–15815.

[80] MacKay DJ. Probable networks and plausible predictions-a review of practical Bayesian methods for supervised neural networks. Network: computation in neural systems. 1995;6(3):469. doi: 10.1088/0954-898X_6_3_011

[81] Immer A, Bauer M, Fortuin V, Rätsch G, Emtiyaz KM. Scalable marginal likelihood estimation for model selection in deep learning. In: International Conference on Machine Learning. PMLR; 2021. p. 4563–4573.

[82] Lotfi S, Izmailov P, Benton G, Goldblum M, Wilson AG. Bayesian model selection, the marginal likelihood, and generalization. In: International Conference on Machine Learning. PMLR; 2022. p. 14223–14247.

[83] Snoek J, Larochelle H, Adams RP. Practical bayesian optimization of machine learning algorithms. Advances in neural information processing systems. 2012;25.

[84] Frazier PI. A tutorial on Bayesian optimization. arXiv preprint arXiv:180702811. 2018;.

[85] Chaloner K, Verdinelli I. Bayesian experimental design: A review. Statistical science. 1995; p. 273–304.

[86] Benda J, Gollisch T, Machens CK, Herz AV. From response to stimulus: adaptive sampling in sensory physiology. Current opinion in neurobiology. 2007;17(4):430–436. doi: 10.1016/j.conb.2007.07.009 17689952

[87] Hock HS, Gordon GP, Whitehurst R. Contextual relations: the influence of familiarity, physical plausibility, and belongingness. Perception & Psychophysics. 1974;16:4–8. doi: 10.3758/BF03203242

[88] Chiao CC, Masland RH. Contextual tuning of direction-selective retinal ganglion cells. Nature neuroscience. 2003;6(12):1251–1252. doi: 10.1038/nn1147 14595442

[89] Fu J, Shrinivasan S, Ponder K, Muhammad T, Ding Z, Wang E, et al. Pattern completion and disruption characterize contextual modulation in mouse visual cortex. bioRxiv. 2023; p. 2023–03.

