# Supplementary material for: Probabilistic neural transfer function estimation with Bayesian system identification
Source: PLoS Comput Biol. 2024 Jul 31;20(7):e1012354. doi: 10.1371/journal.pcbi.1012354 (PMC11318871; doi:10.1371/journal.pcbi.1012354)
Supplement: S1 Appendix — (PDF) [file pcbi.1012354.s001.pdf]

For each CNN model, we tested different numbers (1-5) of blocks (each consists of a convolutional layer and a ReLU layer) and different channel numbers (8, 16, 24, 32, 40 and 48) of each convolutional layer, and selected the ones which yielded (near) optimal predictive performance on validation data. We used small numbers when the performance was similar across models. For each dataset, the six methods used similar model architecture with the baseline CNN, except that the dropout model had dropout layers after the two ReLU functions and the FC layer.

To estimate the uncertainty of neuronal responses for a trained probabilistic model, We ran the model prediction for 100 sampling times. We defined variance of predicted response for one neuron as $Response\ variance = E[Var[D]_s]_n$ (sampling times s, test stimulus number n, and response matrix D with a shape of $s \times n$). The overall response variance for a model was an average of response variances for the recorded neurons. To calculate the variance of recorded response for a neuron, we replaced the sampling times with the repeated times of the presented test stimulus.
