# Supplementary figures and images for: Probabilistic neural transfer function estimation with Bayesian system identification
Source: PLoS Comput Biol. 2024 Jul 31;20(7):e1012354. doi: 10.1371/journal.pcbi.1012354 (PMC11318871; doi:10.1371/journal.pcbi.1012354)

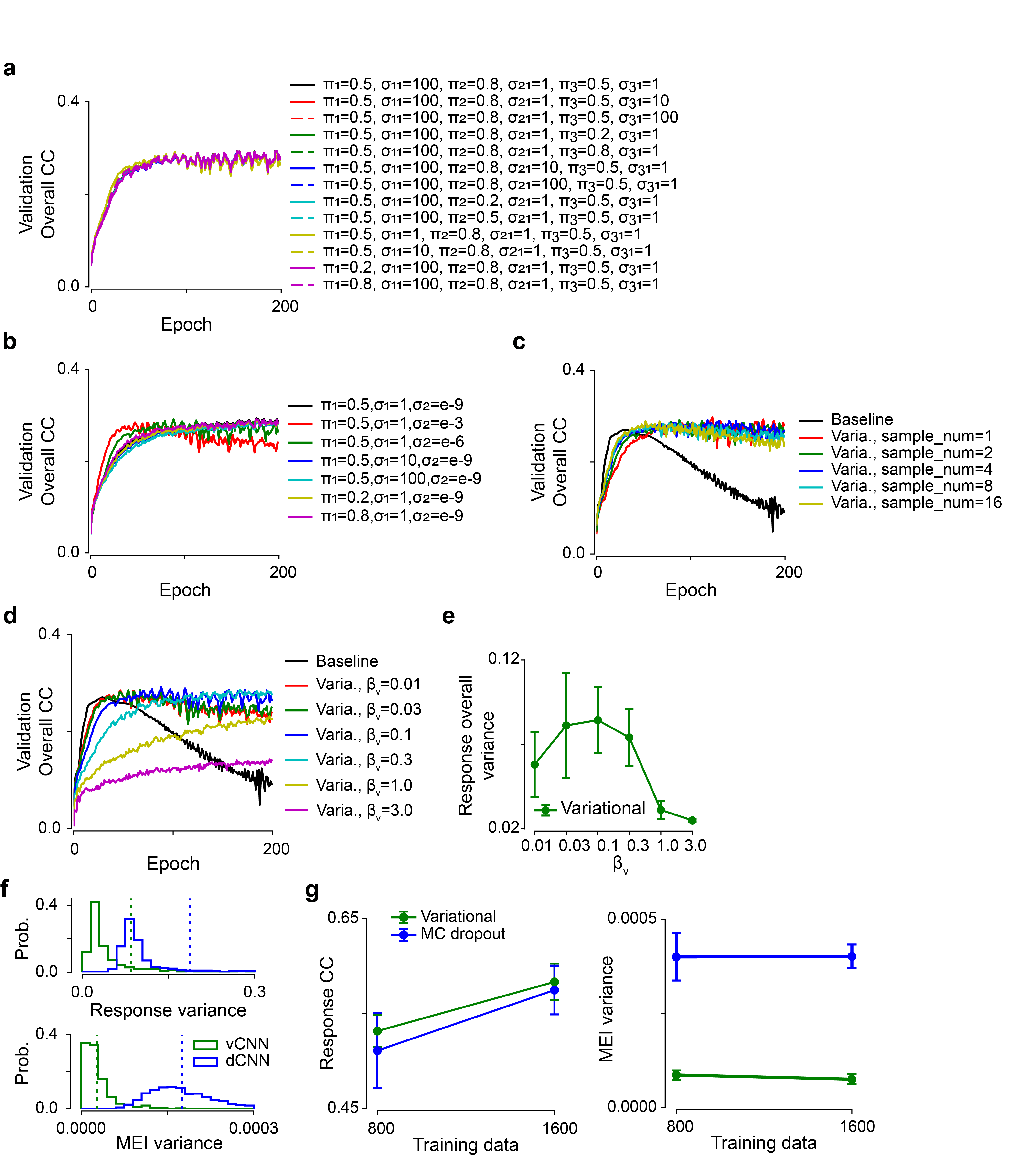

Supplement: S1 Fig — (a,b) Predictive performance (correlation coefficient, CC) based on validation data during training for variational models (βv = 0.1) with different prior distributions. All layers adopted the same σ2 = exp(−6) with different π and σ1 values (a), or with the same parameters of prior distribution (b). We picked π = 0.5, σ1 = 1, σ2 = exp(−6) for subsequent model training. (c) Predictive performance based on validation data during model training for different numbers of Monte Carlo sampling. We picked number = 1 or 2 to save training time. (d) Model performance based on validation data during training for the baseline and variatonal models with different βv values. (e) Overall variance of predicted responses to test stimuli for different βv values. (f) Histogram of response variance (top) and MEI (RF) variance (bottom) for the variational and the MC dropout models. Dotted line represents the mean of histogram. (g) Model performance (left) based on test data and RF overall variance (right) for two probabilistic models with different amounts of training data. Error bars in (e) and (g) represent standard deviation of n = 10 random seeds for each model. (TIF) [file pcbi.1012354.s002.tif]

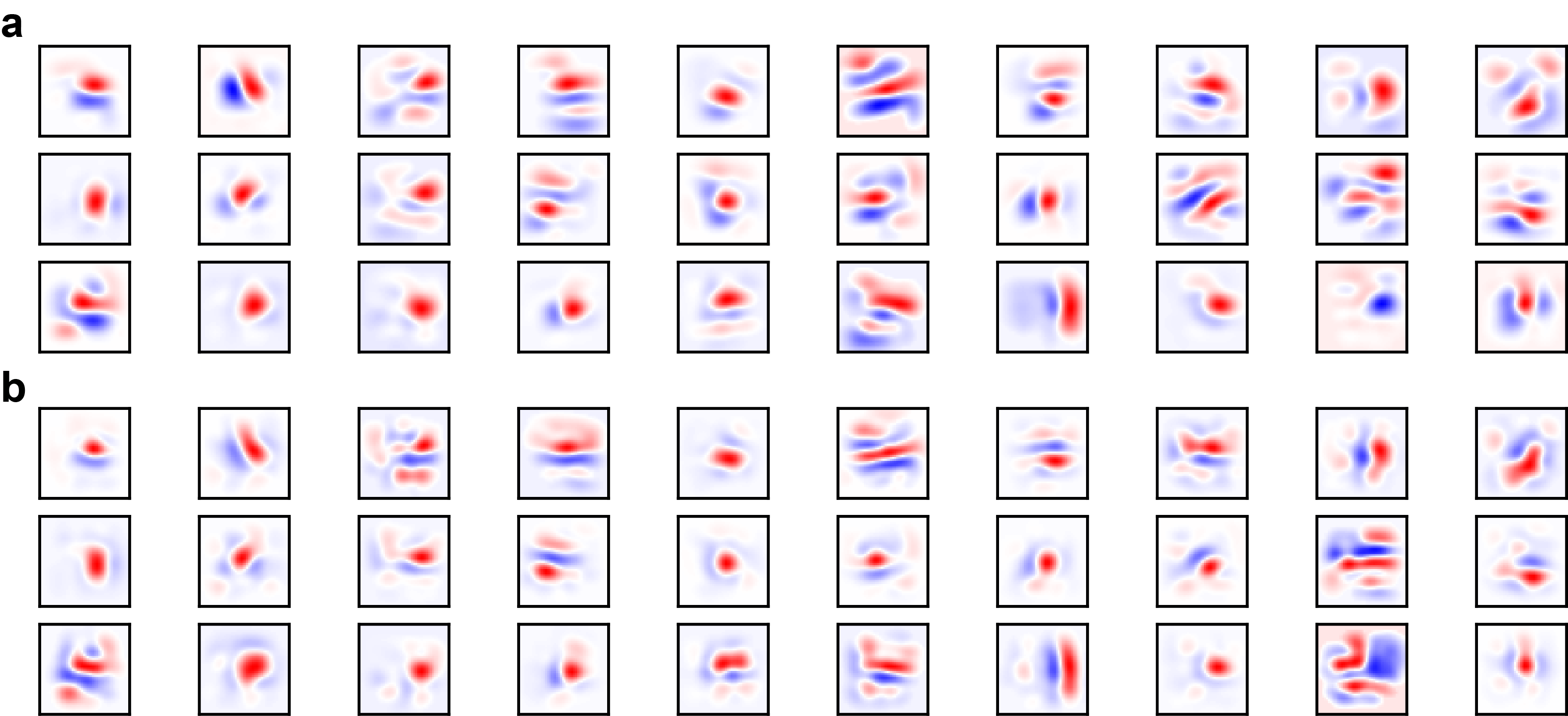

Supplement: S2 Fig — (a,b) MEIs of 30 exemplary neurons for the first dataset generated by the L2+L1 model (a) and the variational one (b). (TIF) [file pcbi.1012354.s003.tif]

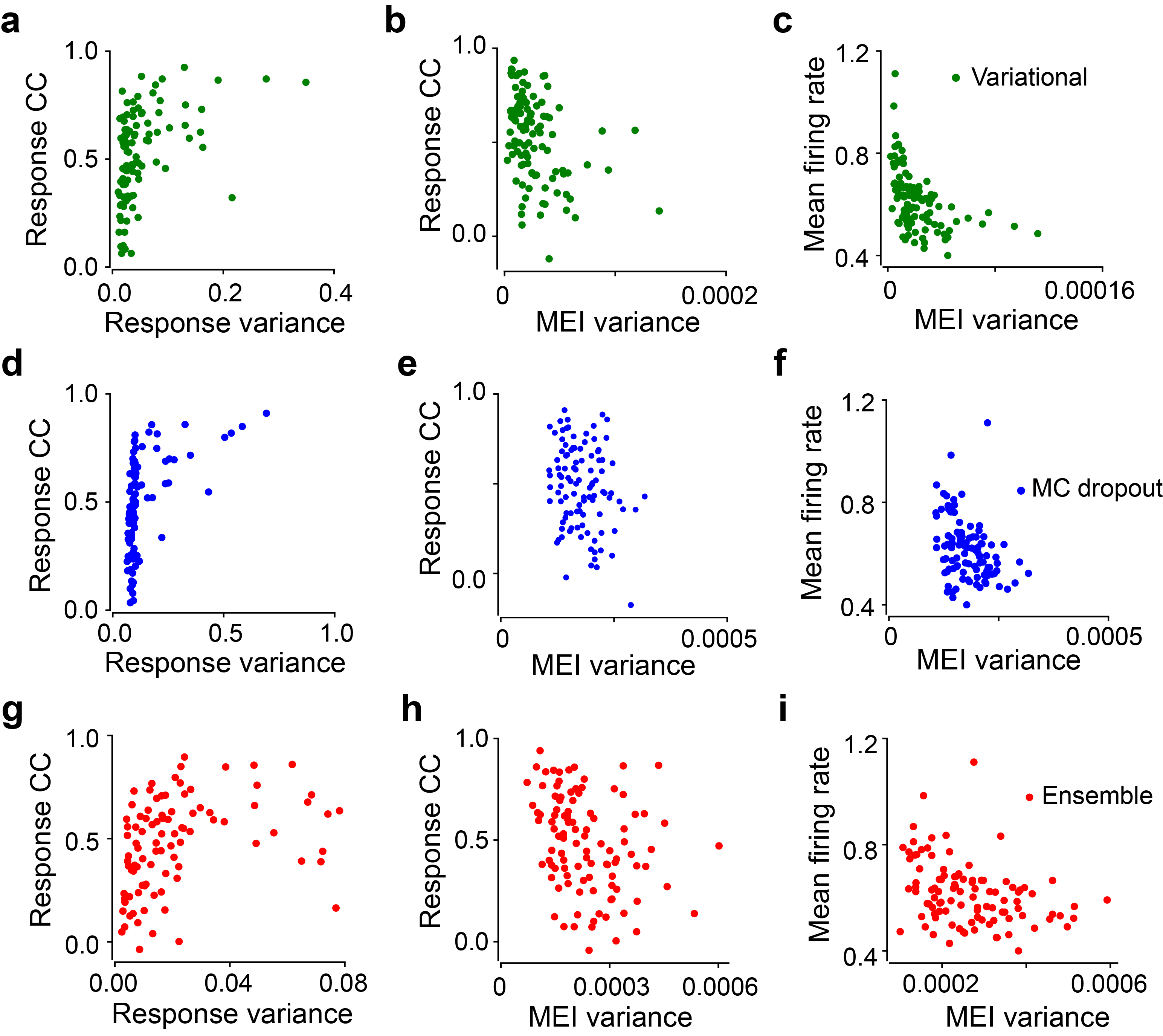

Supplement: S3 Fig — (a) Scatter plot of response CC and response variance for variational model at one random seed (each dot representing one neuron; CC = 0.24, p = 0.05). (b) Scatter plot of response CC and MEI variance for variational model at one random seed (each dot representing one neuron; CC = −0.37, p = 0.0001). (c) Scatter plot of mean firing rate and MEI variance for variational model at one random seed (each dot representing one neuron; CC = −0.47, p < 0.0001). (d) Same with (a), but for MC dropout model (CC = 0.18, p = 0.07). (e) Same with (b), but for MC dropout model (CC = −0.23, p = 0.02). (f) Same with (c), but for MC dropout model (CC = −0.33, p = 0.006). (g) Same with (a), but for ensemble model (CC = 0.19, p = 0.06). (h) Same with (b), but for ensemble model (CC = −0.34, p = 0.0004). (i) Same with (c), but for ensemble model (CC = −0.35, p = 0.0003). (TIF) [file pcbi.1012354.s004.tif]

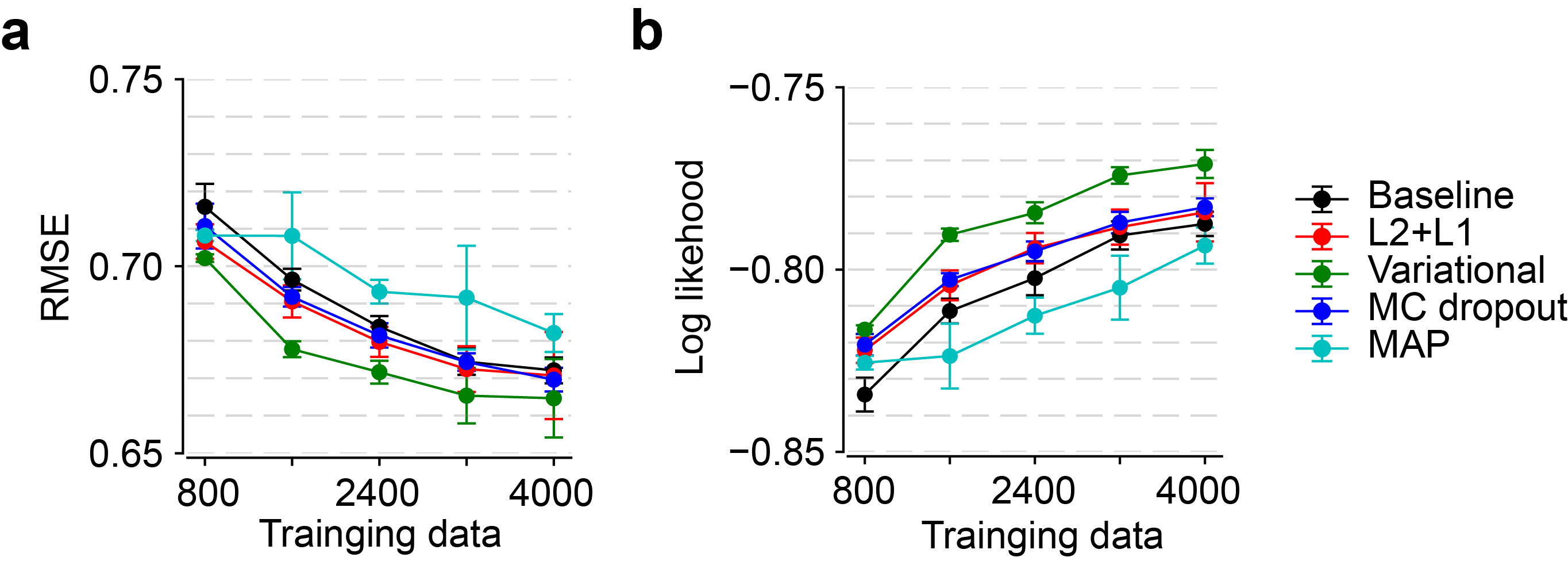

Supplement: S4 Fig — (a,b) Like Fig 5a but using RMSE (a) and log likelihood (b) to compare models. When using RMSE, we found our variational method had equivalent prediction accuracy to the MC dropout model in the condition of full data (p = 0.1709), and the Bayesian one outperformed the MC dropout one in conditions of less data (p = 0.0001 at 20% of data, p < 0.0001 at 40%, p = 0.0001 at 60%, p = 0.0021 at 80%). When using log likelihood, the variational model had significantly higher predictive performance than the MC dropout method (p = 0.0002 at 20%, p < 0.0001 at 40%, p < 0.0001 at 60%, p < 0.0001 at 80%, p < 0.0001 at 100%). (TIF) [file pcbi.1012354.s005.tif]

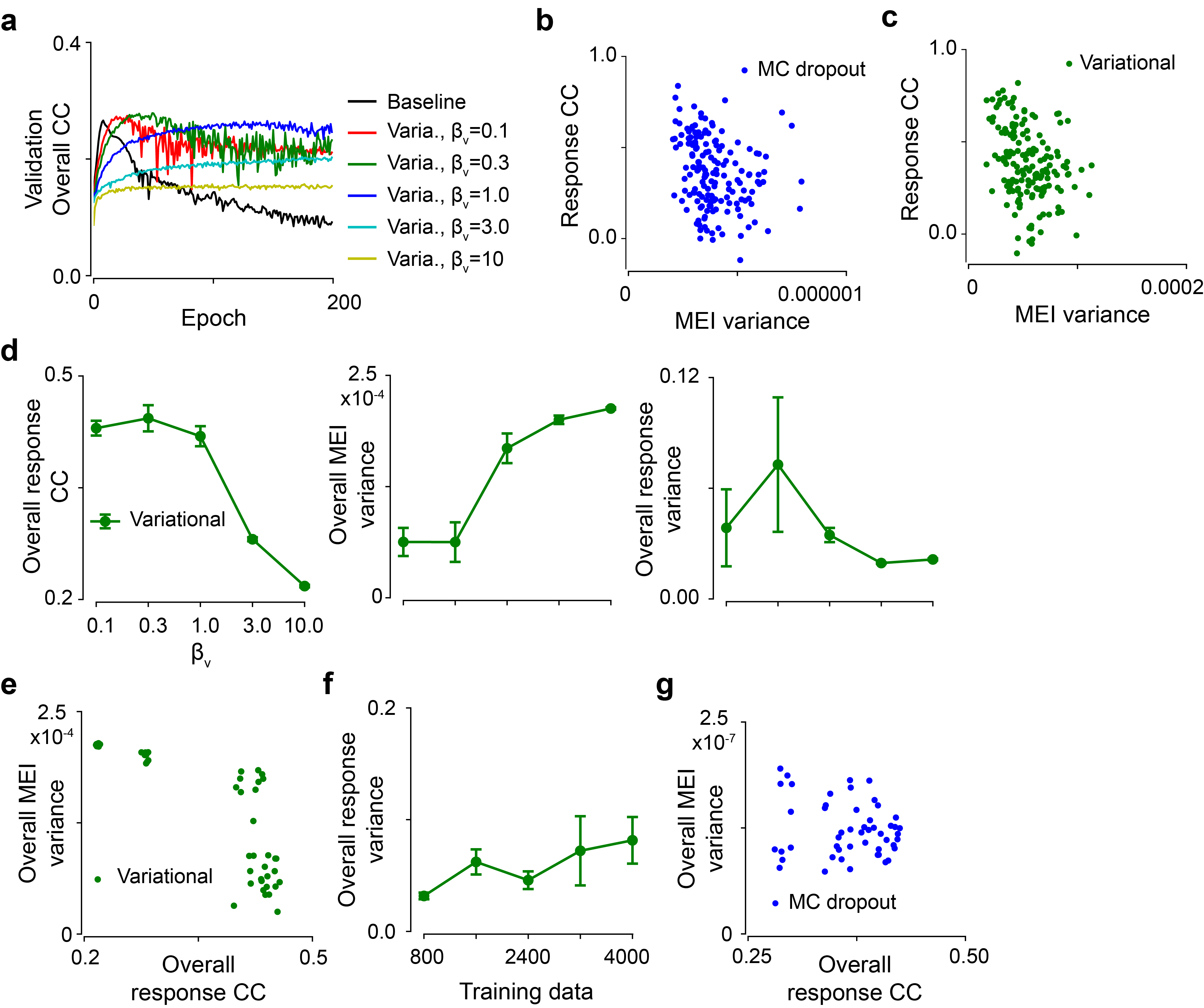

Supplement: S5 Fig — (a) Model performance based on validation data during training for the baseline and the variational models with different βv values. (b,c) Scatter plot of response CC and MEI (RF) variance for MC dropout (b) and variational (c) models for 10 seeds (CC = −0.25, p = 0.001 and CC = −0.34, p < 0.0001 for dropout and variational one, each dot representing one neuron at one random seed). (d) Predictive performance, overall RF variance and overall response variance for variational models with different βv values. (c) Predictive performance based on validation data during model training for different numbers of Monte Carlo sampling. We picked number = 1 or 2 to save training time. (d) Model performance based on validation data during training for the baseline and the variational ones with different βv values. (e) Scatter plot for overall response CC and overall RF variance for the variational methods with different βv values (d) and at 10 seeds (CC = −0.82, p < 0.0001). Each dot represents one model. (f) Overall response variance for different amounts of training data for the variational models (10 seeds per model). (g) Scatter plot for overall response CC and overall RF variance for the dropout model with different amounts of training data and at 10 seeds (CC = −0.17, p = 0.24). Each dot represents one model. Error bars in (d) and (f) represent standard deviation of n = 10 random seeds for each model. (TIF) [file pcbi.1012354.s006.tif]

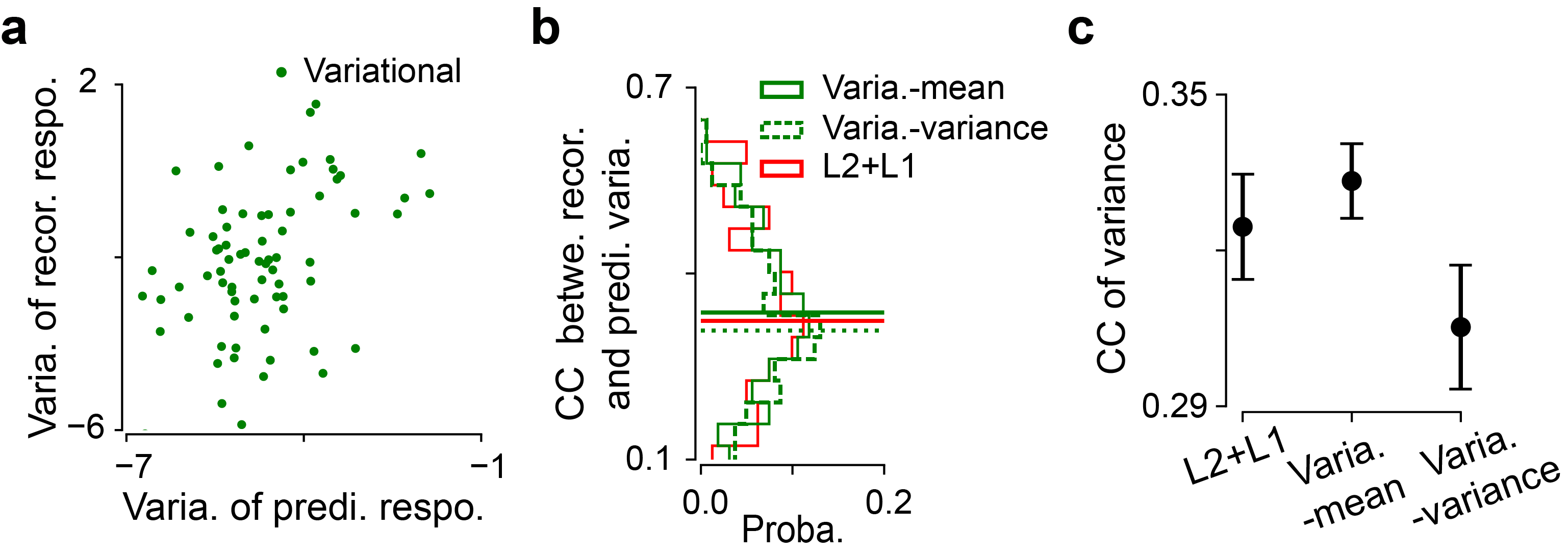

Supplement: S6 Fig — Using the trained models, we tested whether the variance of predicted responses was related to the variance of recorded responses for each neuron. We first estimated the predicted response variance to a stimulus. For the L2+L1 model, as the mean of neural responses is proportional to the variance, we used the model output (a single predicted value) as a substitute. For the variational one, we either used the mean of predicted responses (multiple sampling times) as a substitute or calculated the response variance explicitly. (a) Scatter plot (axes in log scale) of predicted response variance (using response mean as a substitute) and recorded response variance for one neuron for a variational model. Each dot representing one stimulus. (b) Distribution of correlations between recorded and predicted response variance for all neurons for the L2+L1, variational-mean (using response mean as a substitute) and variational-variance (calculating response variance), at one random seed. Horizontal lines representing distribution means. (c) Mean correlations between two response variances (10 seeds per model). Note that variational-variance had lower correlation than the L2+L1. Error bars represent standard deviation of n = 10 random seeds for each model. We computed the correlation using the predicted and recorded response variances of the test stimuli for each neuron (CC = 0.34, p = 0.002, Spearman correlation for an exemplary neuron; a). We found that the variational one using response mean as a substitute of variance had a slightly higher mean correlation across neurons compared to the L2+L1 (p = 0.0368, two-sided permutation tests on 10 random seeds for 10,000 times; b,c). (TIF) [file pcbi.1012354.s007.tif]

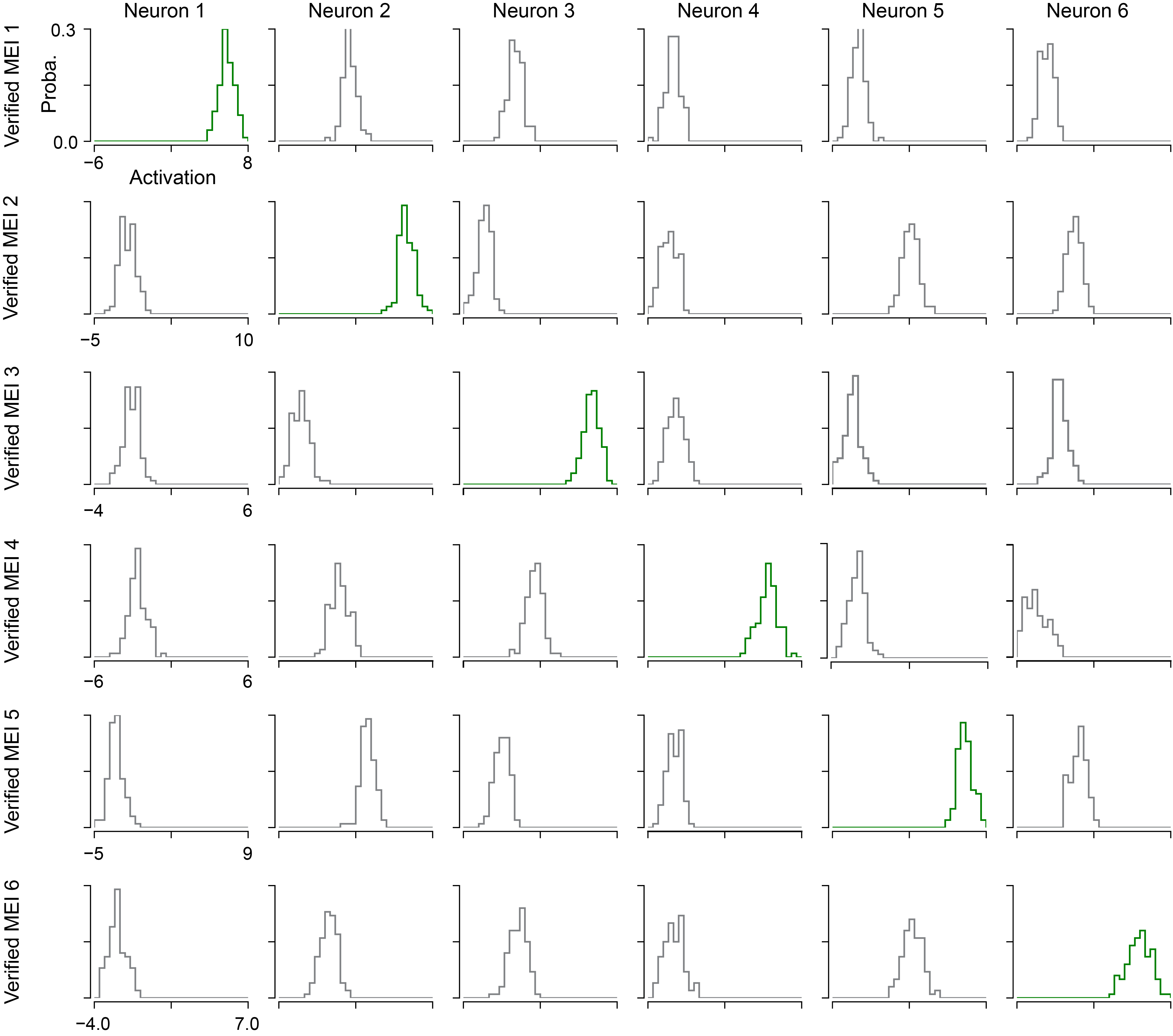

Supplement: S7 Fig — Instead of performing closed-loop experiments to examine the effectiveness of our veriational model, we used the verified MEIs from previous study to compare the neuronal activities driven by different stimuli [21]. We plotted 1D histograms of activation of 6 exemplary cells (from left to right) driven by the verified MEIs (from top to bottom). We used green color instead of gray to highlight the highest neuronal activation on the diagonal (driven by the respective verified MEI). (TIF) [file pcbi.1012354.s008.tif]

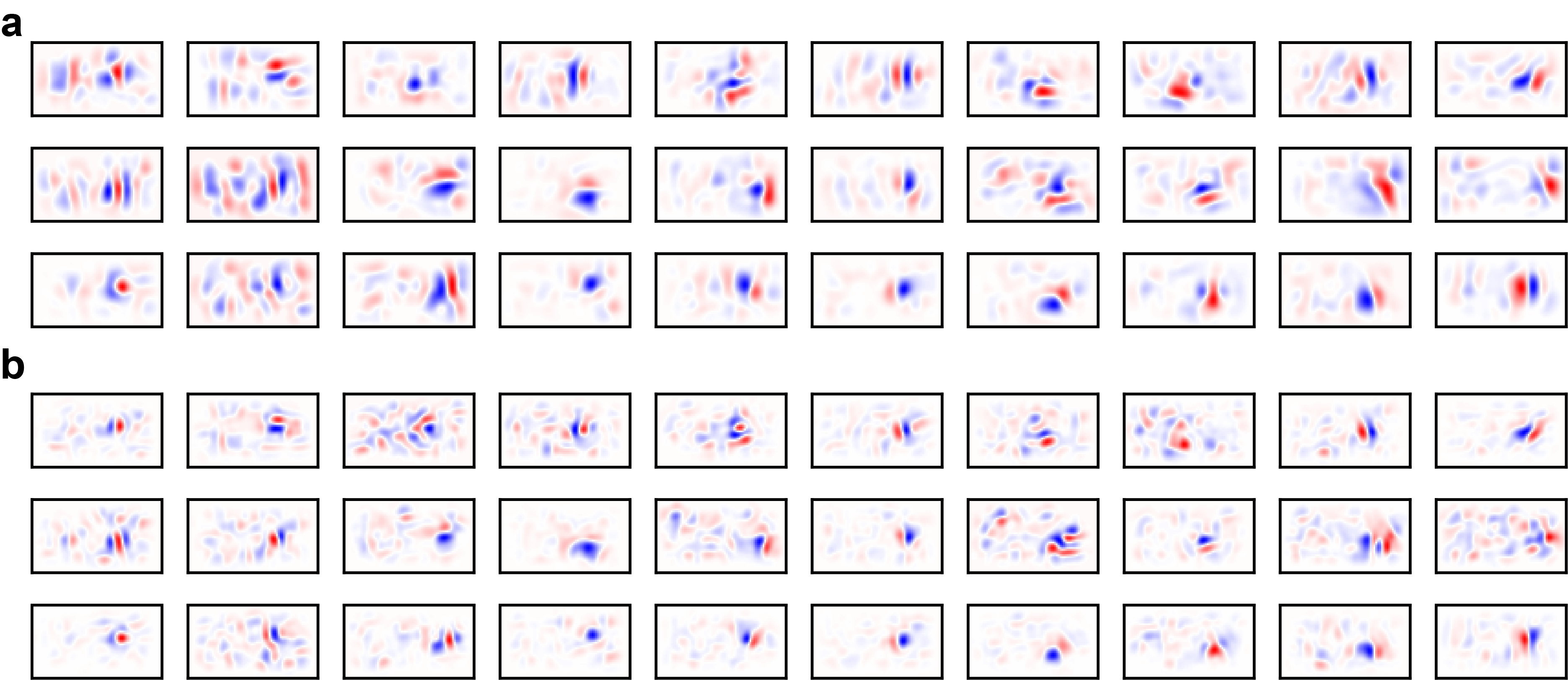

Supplement: S8 Fig — (a,b) MEIs in the UV channel of 30 exemplary neurons generated by L2+L1 model (a) and variational one (b). (TIF) [file pcbi.1012354.s009.tif]
